# Supplementary material for: Systematic in silico discovery of novel solute carrier-like proteins from proteomes
Source: PLoS One. 2022 Jul 28;17(7):e0271062. doi: 10.1371/journal.pone.0271062 (PMC9333335; doi:10.1371/journal.pone.0271062)

Species tree:

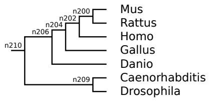

SLC1 family

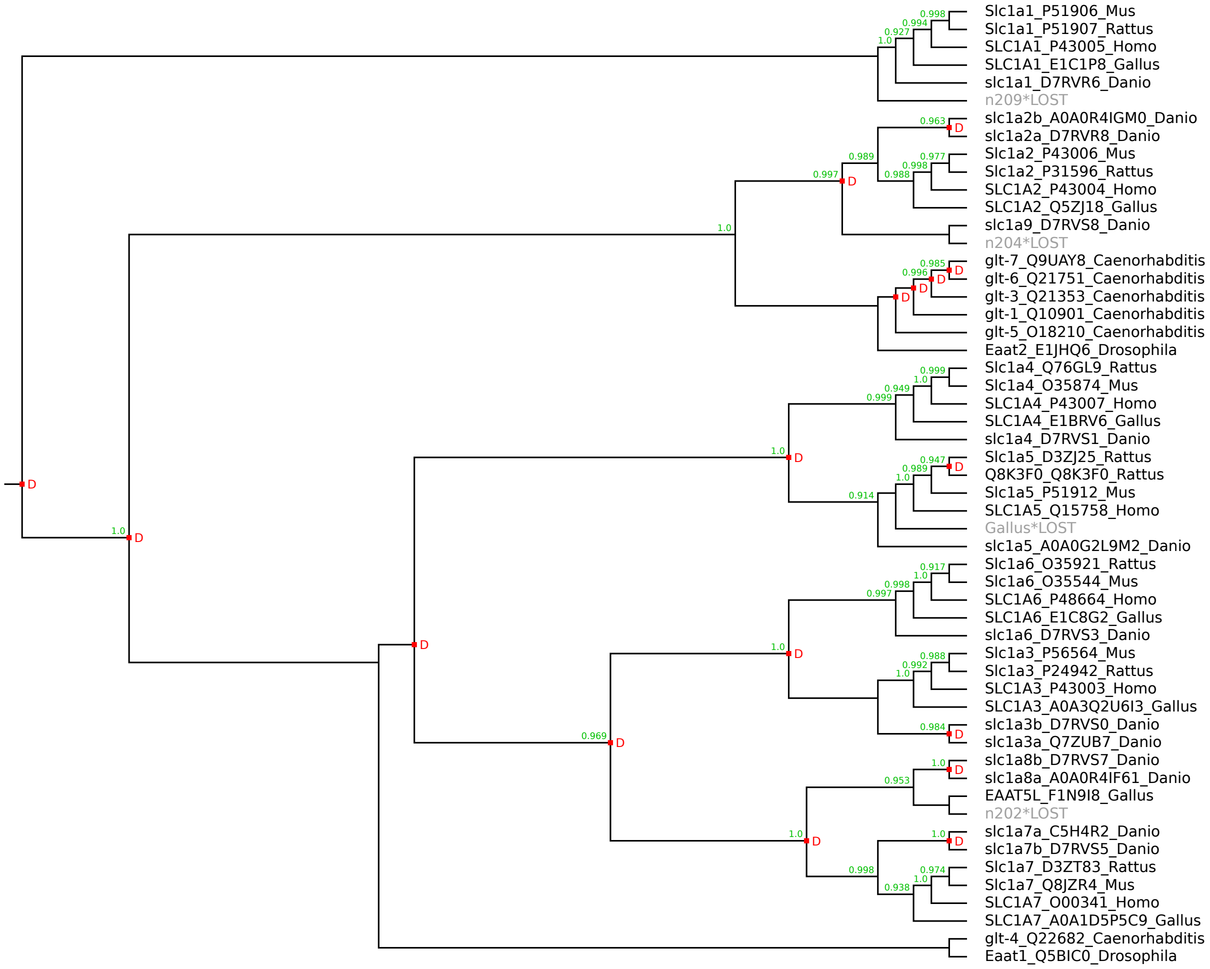

Species tree:

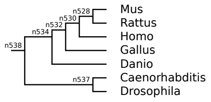

SLC2 family

Warning: this is a large figure that had to be reduced to fit on the page.
Please use the zoom function of your PDF viewer to see the details.

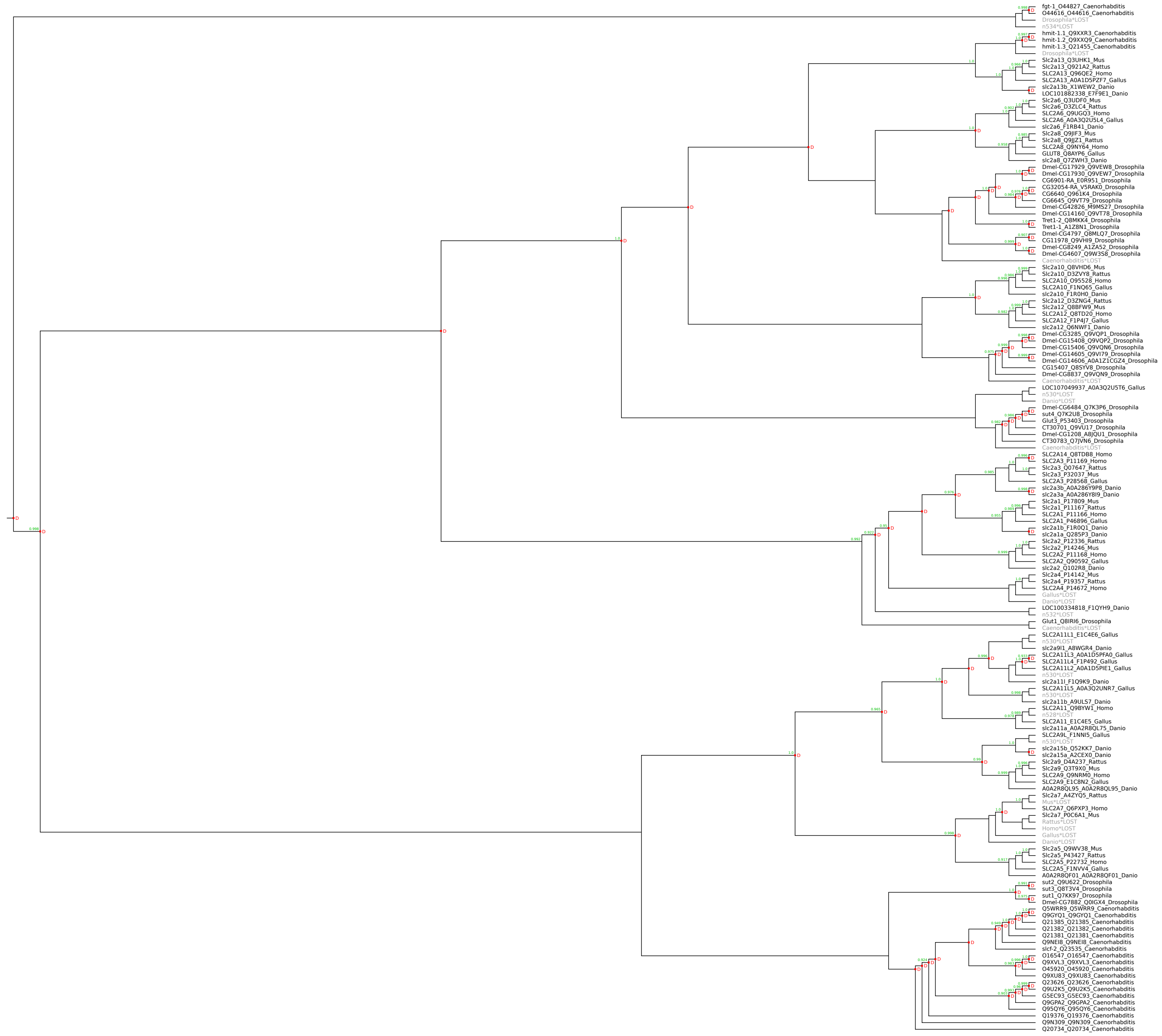

Species tree:

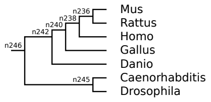

SLC4 family

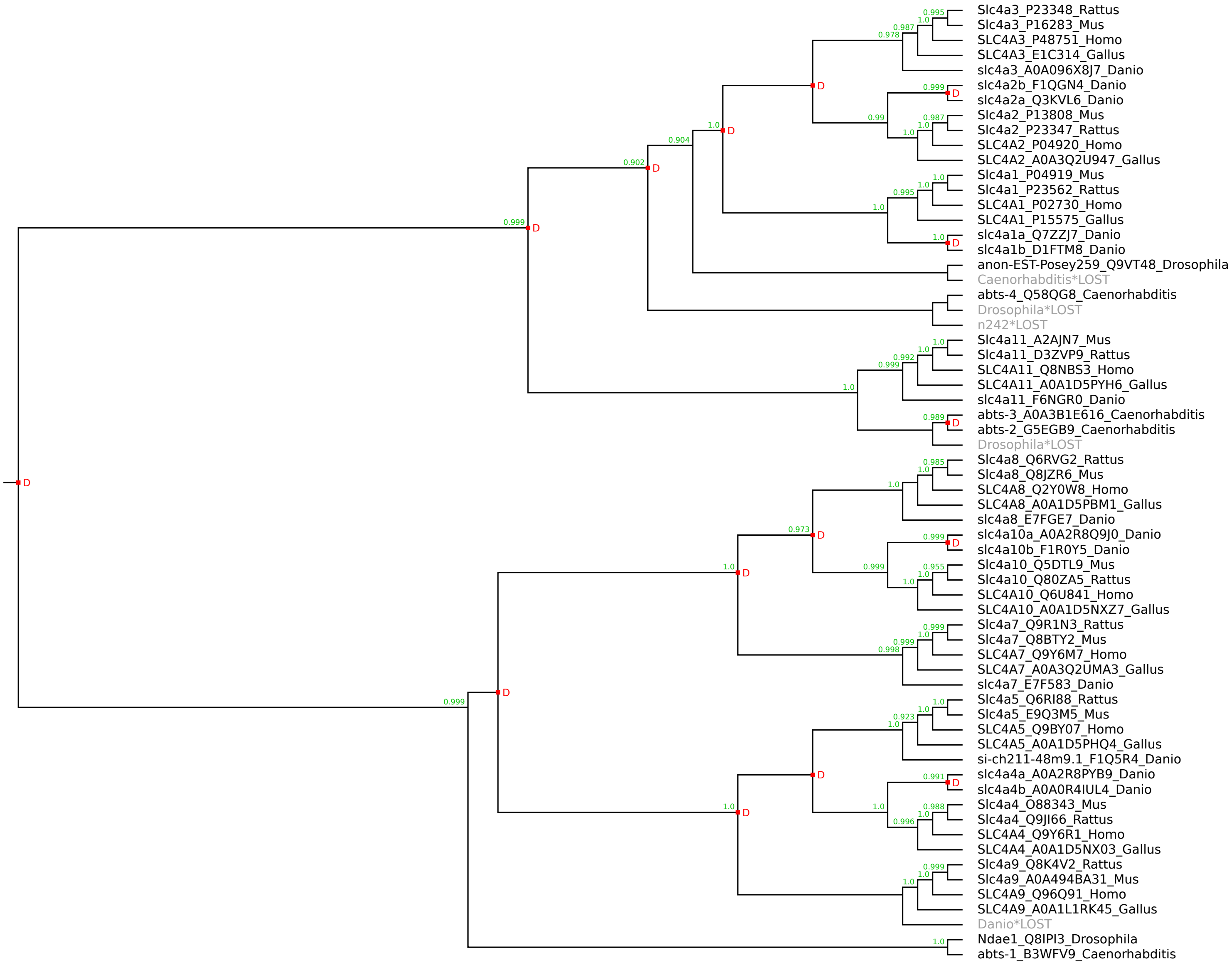

SLC5 family

Species tree:

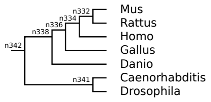

Warning: this is a large figure that had to be reduced to fit on the page.
Please use the zoom function of your PDF viewer to see the details.

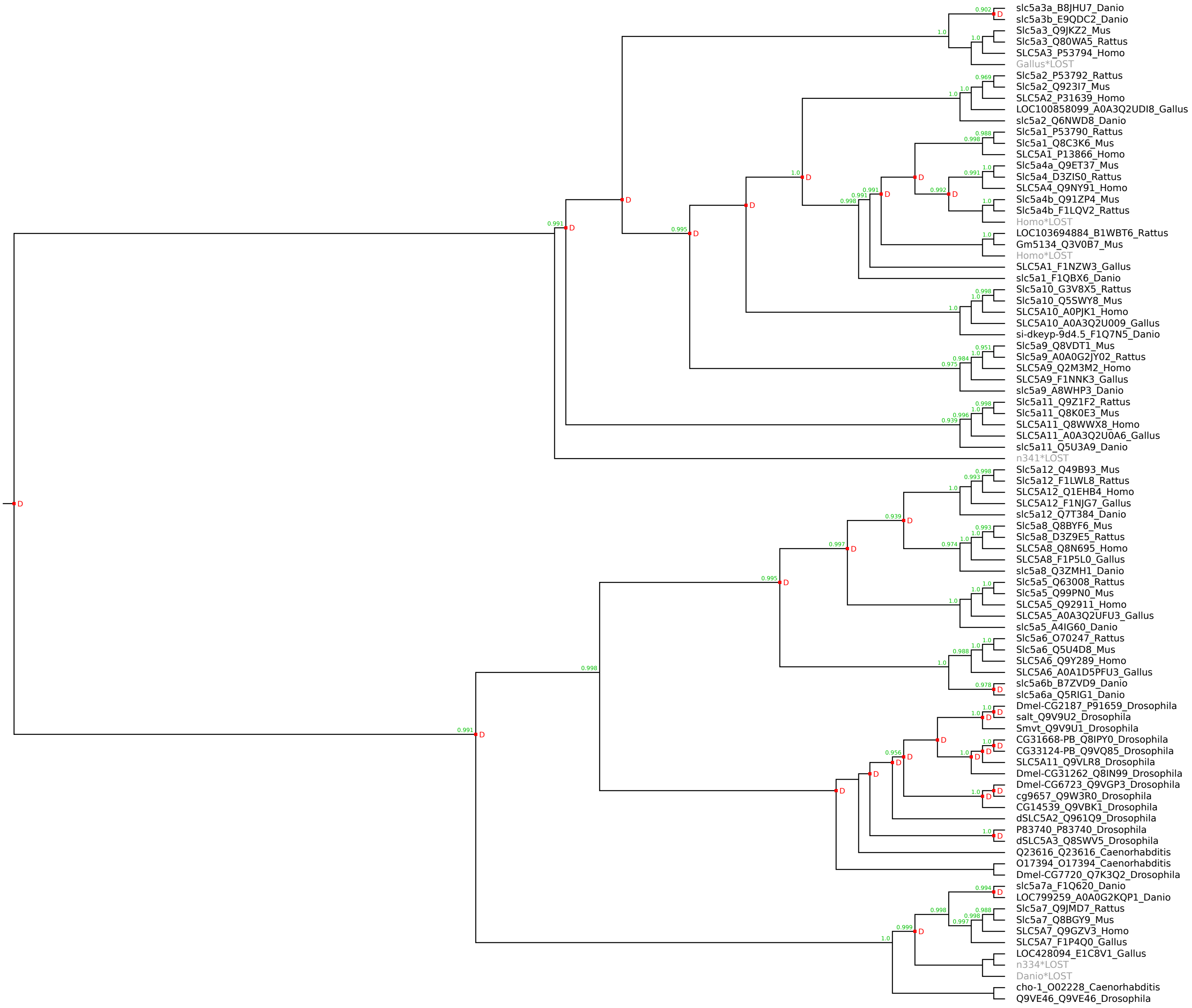

Species tree:

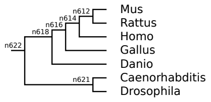

SLC6 family

Warning: this is a large figure that had to be reduced to fit on the page.
Please use the zoom function of your PDF viewer to see the details.

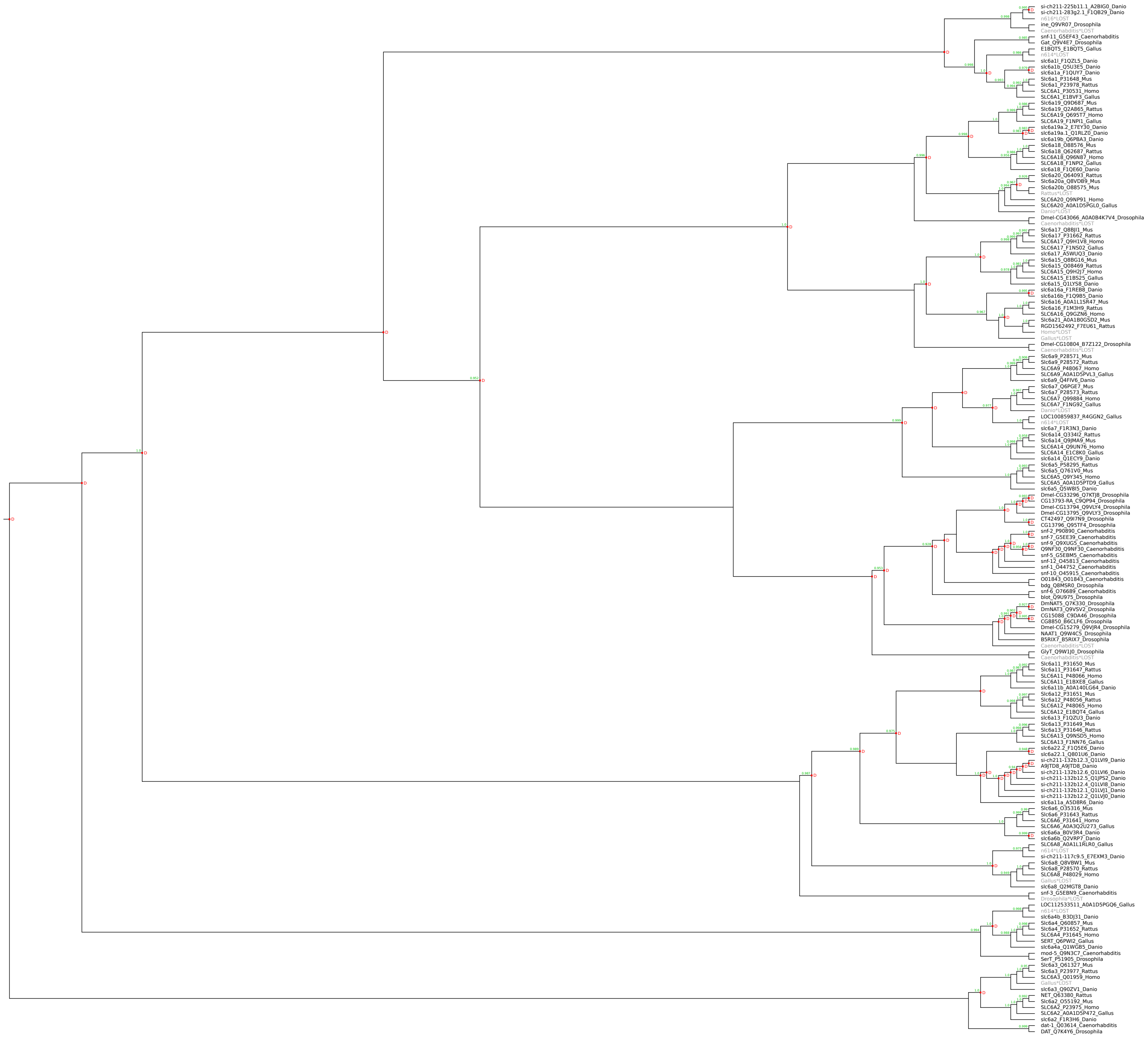

SLC7 family

Species tree:

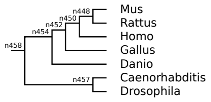

Warning: this is a large figure that had to be reduced to fit on the page.
Please use the zoom function of your PDF viewer to see the details.

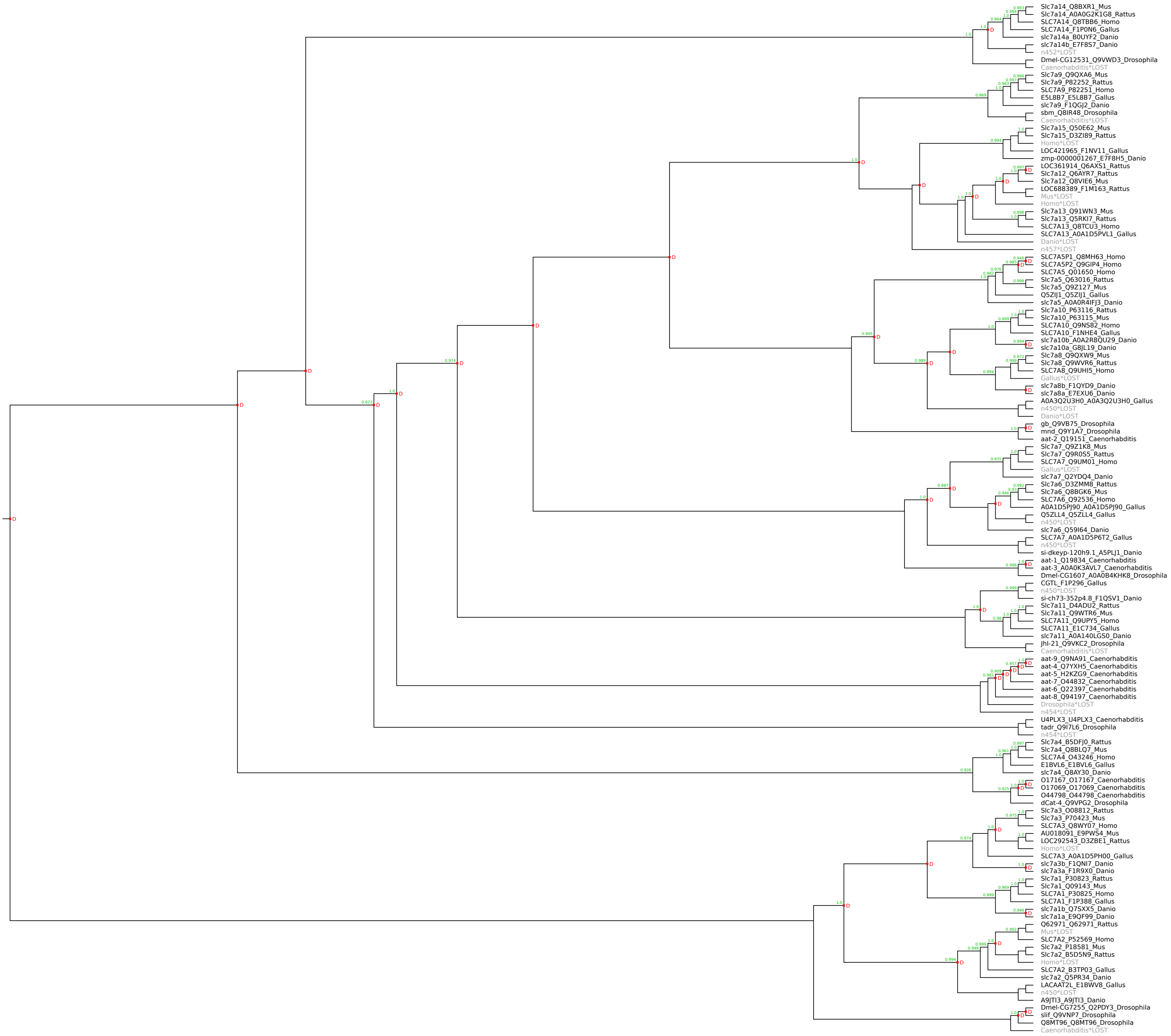

SLC8 family

Species tree:

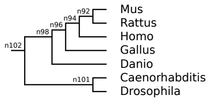

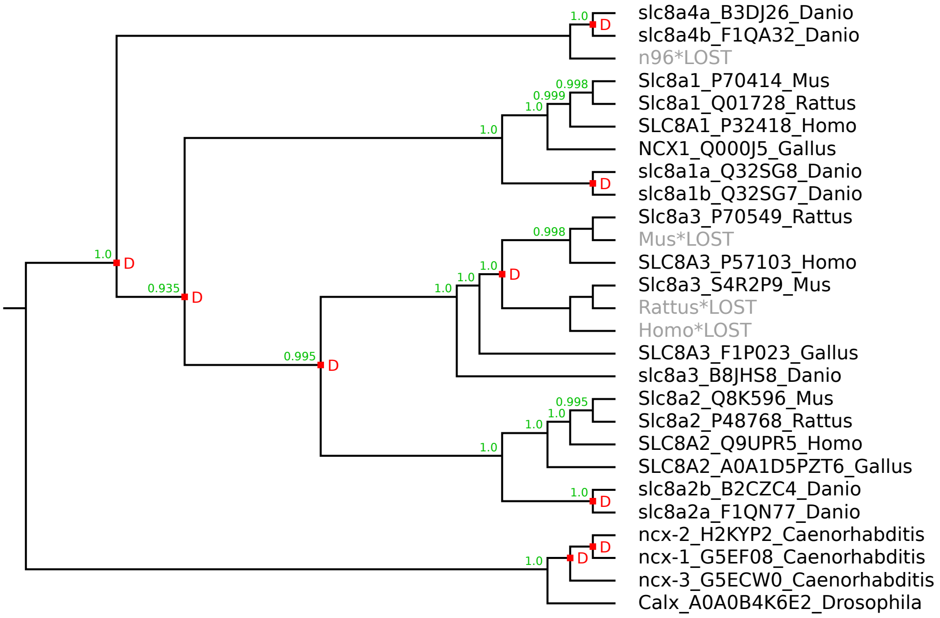

SLC9 family

Species tree:

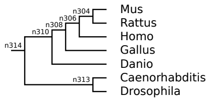

Warning: this is a large figure that had to be reduced to fit on the page.
Please use the zoom function of your PDF viewer to see the details.

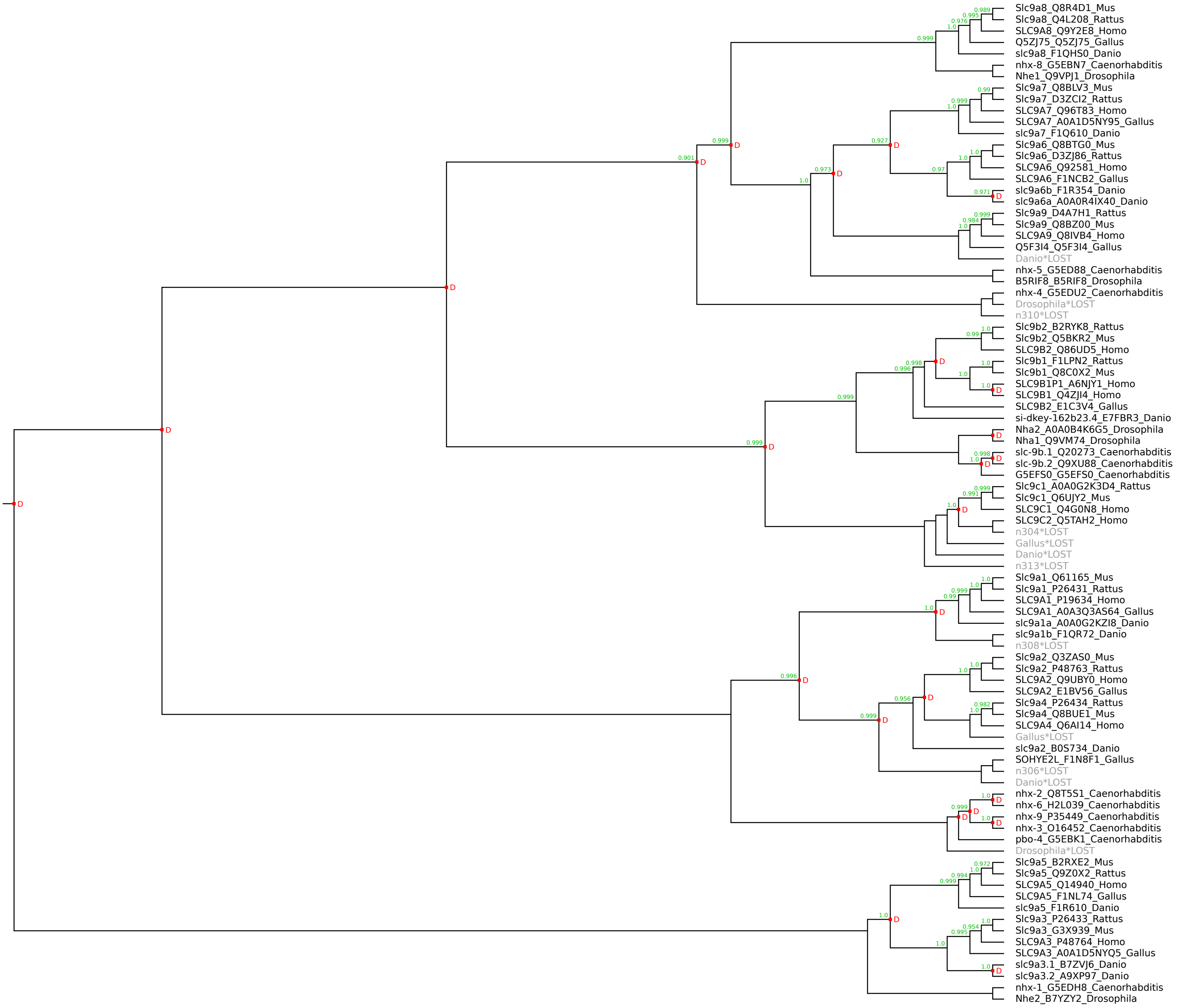

SLC10 family

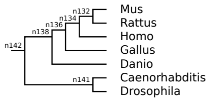

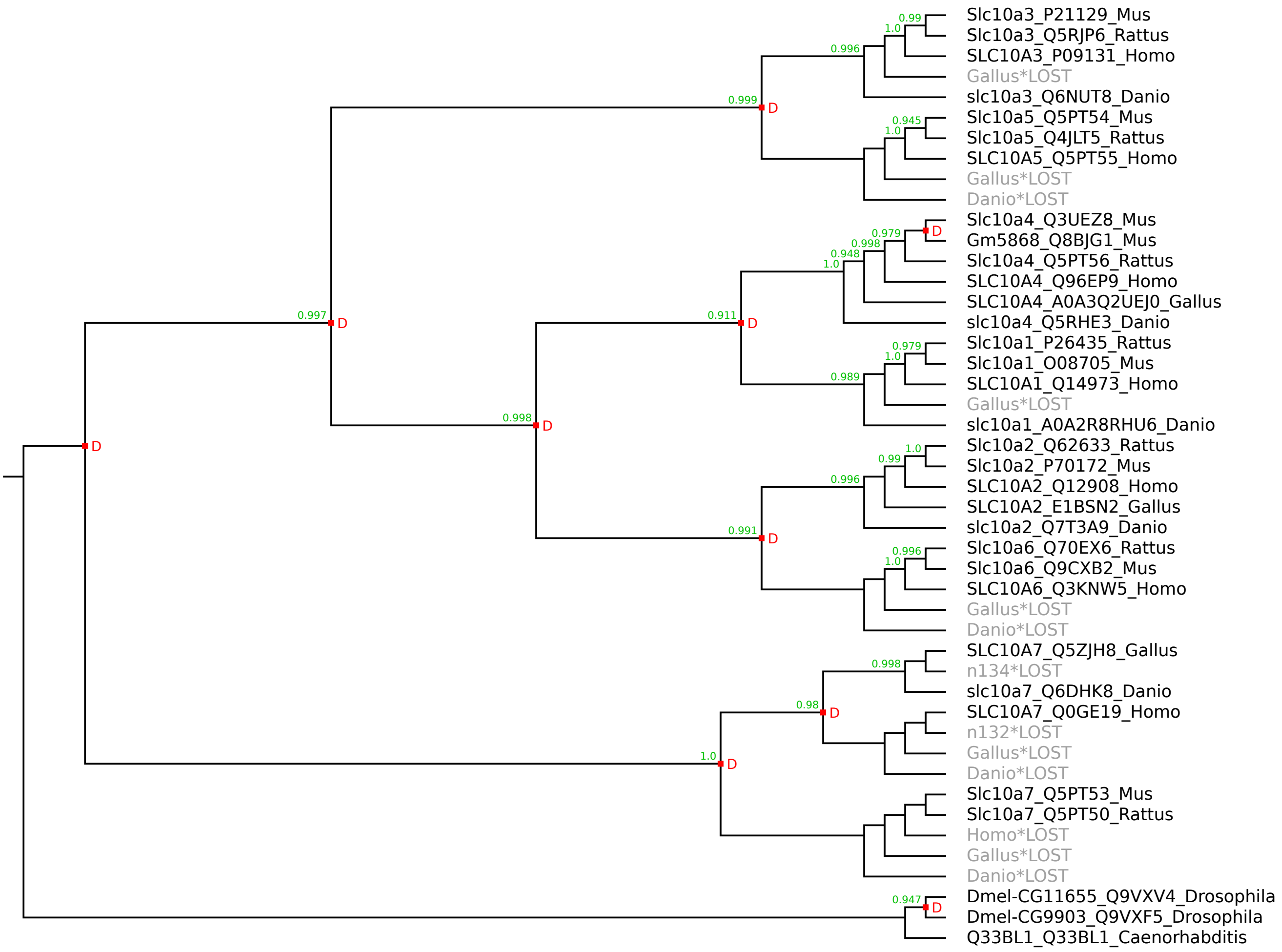

SLC11 family

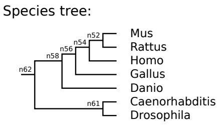

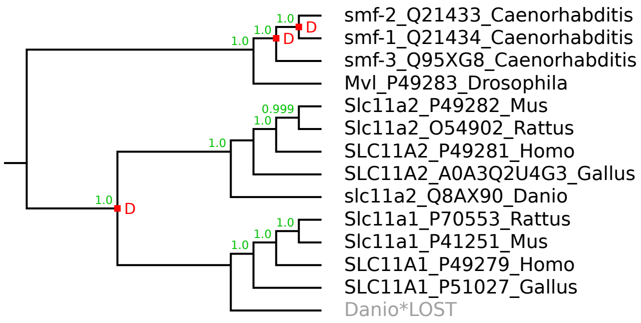

SLC12 family

Species tree:

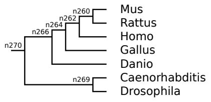

Warning: this is a large figure that had to be reduced to fit on the page.
Please use the zoom function of your PDF viewer to see the details.

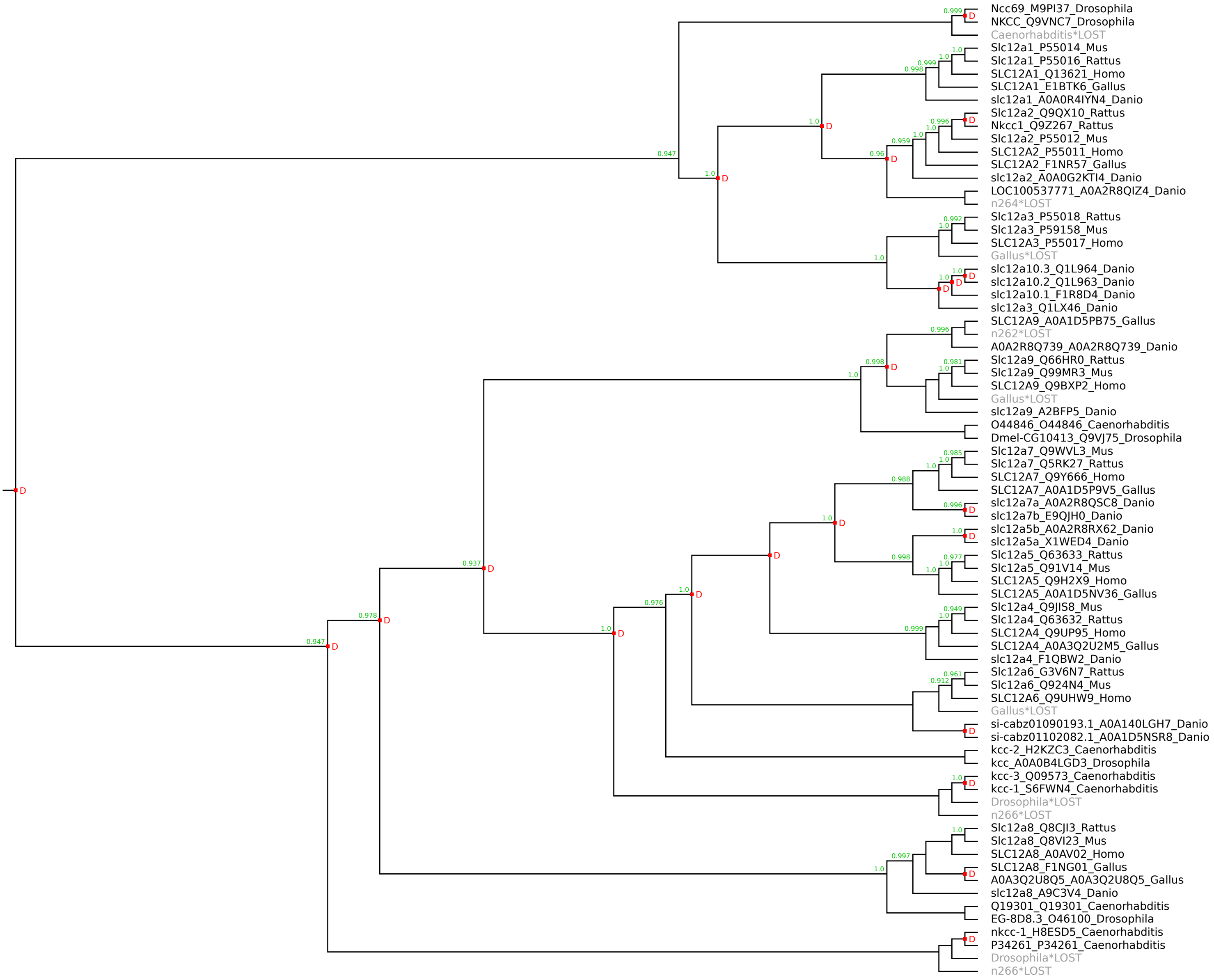

SLC13 family

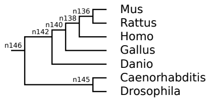

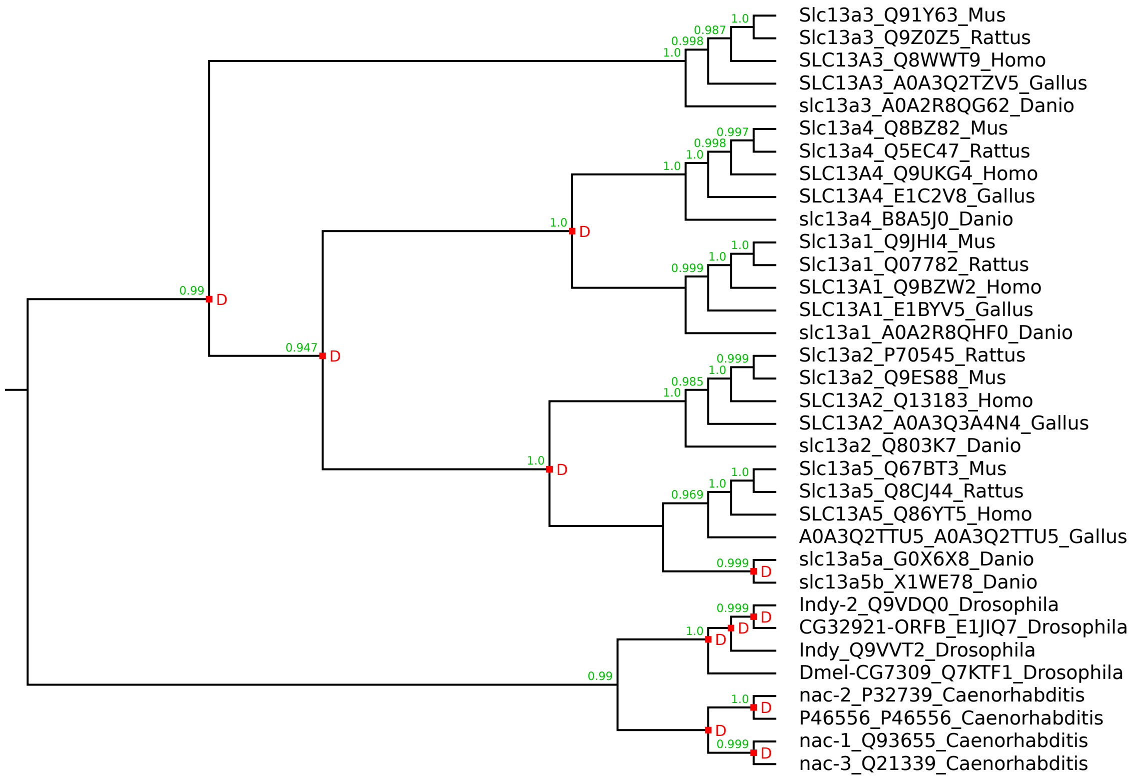

SLC14 family

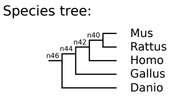

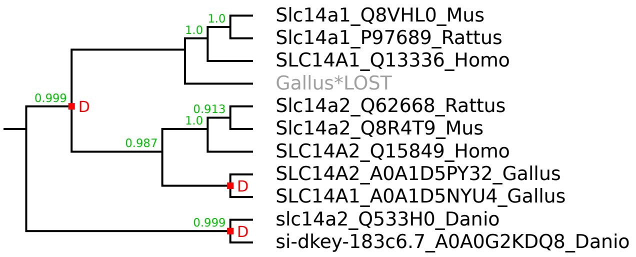

SLC15 family

Species tree:

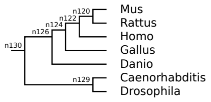

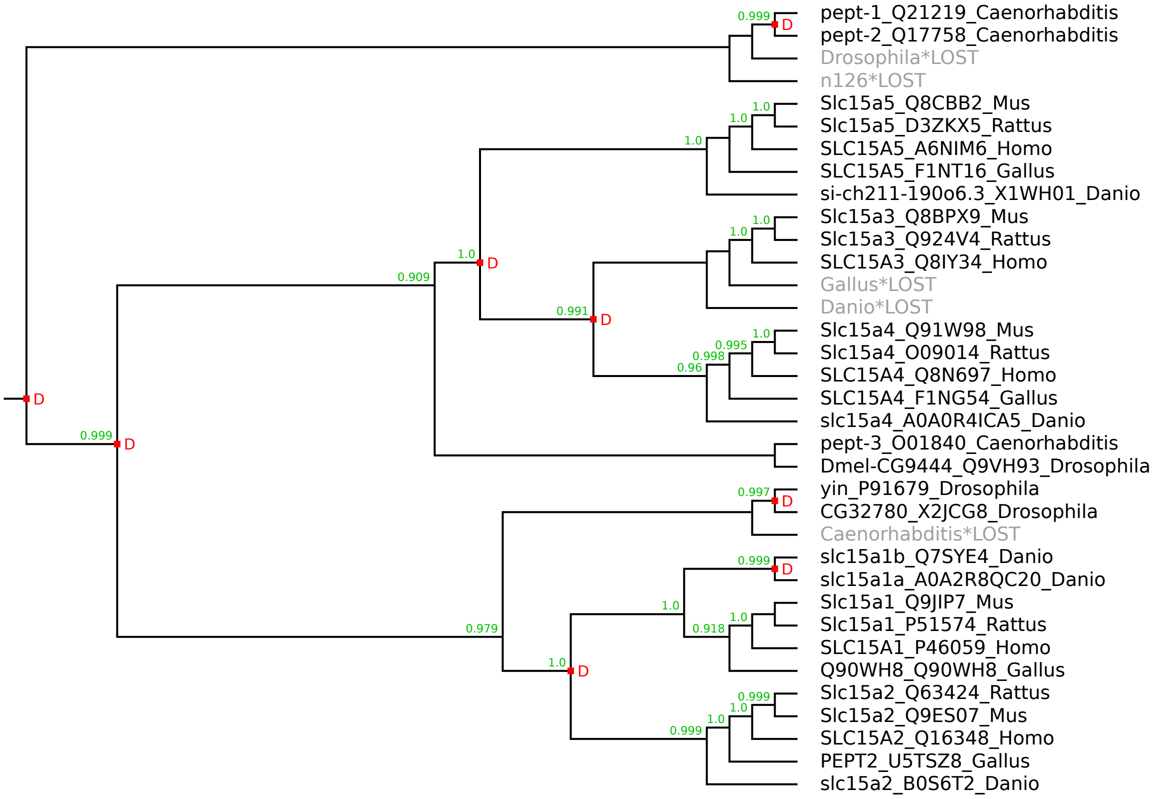

SLC16 family

Species tree:

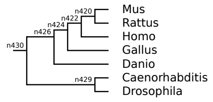

Warning: this is a large figure that had to be reduced to fit on the page.
Please use the zoom function of your PDF viewer to see the details.

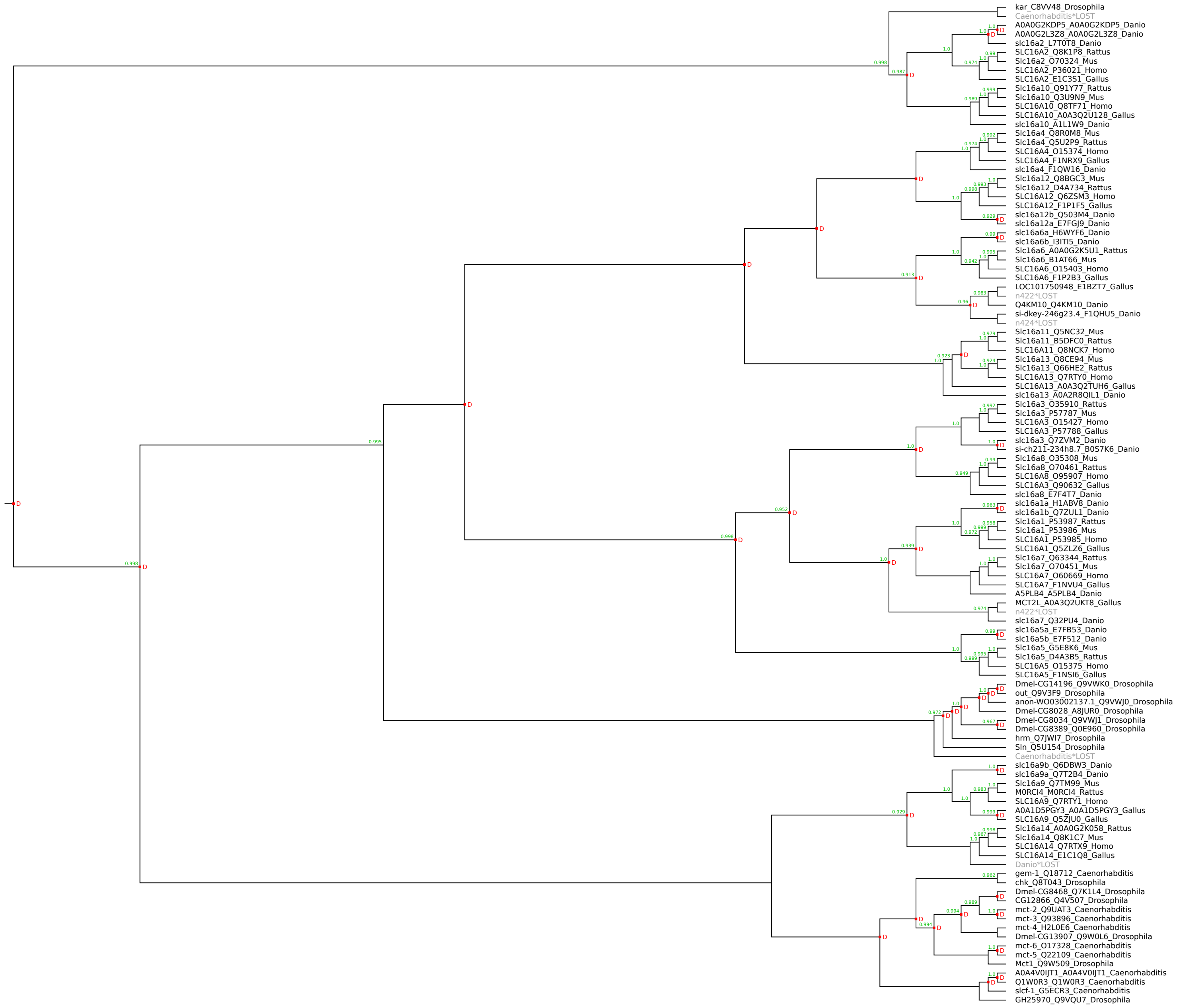

SLC17 family

Species tree:

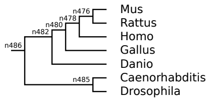

Warning: this is a large figure that had to be reduced to fit on the page.
Please use the zoom function of your PDF viewer to see the details.

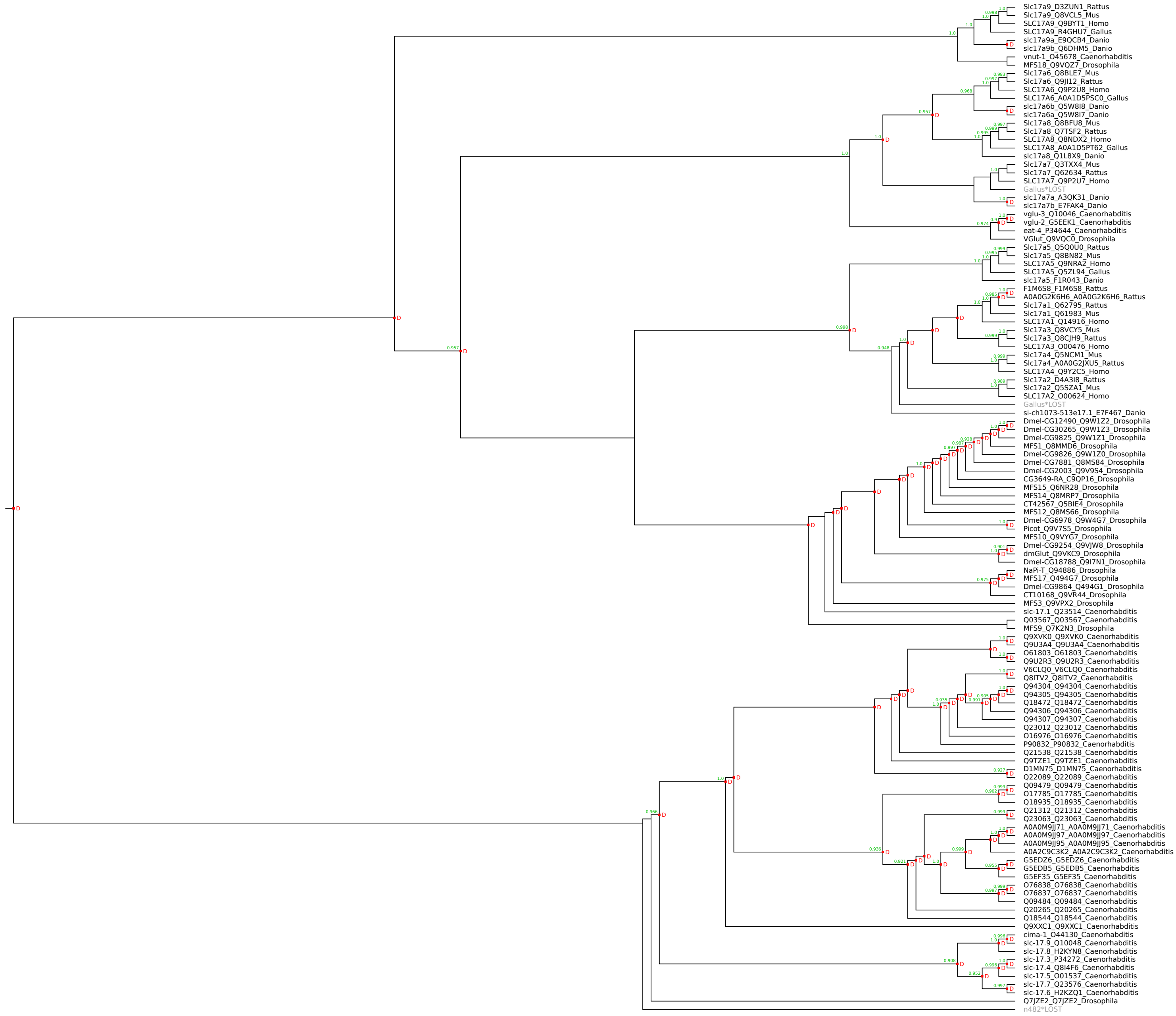

SLC18 family

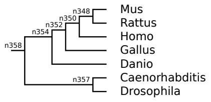

Warning: this is a large figure that had to be reduced to fit on the page.
Please use the zoom function of your PDF viewer to see the details.

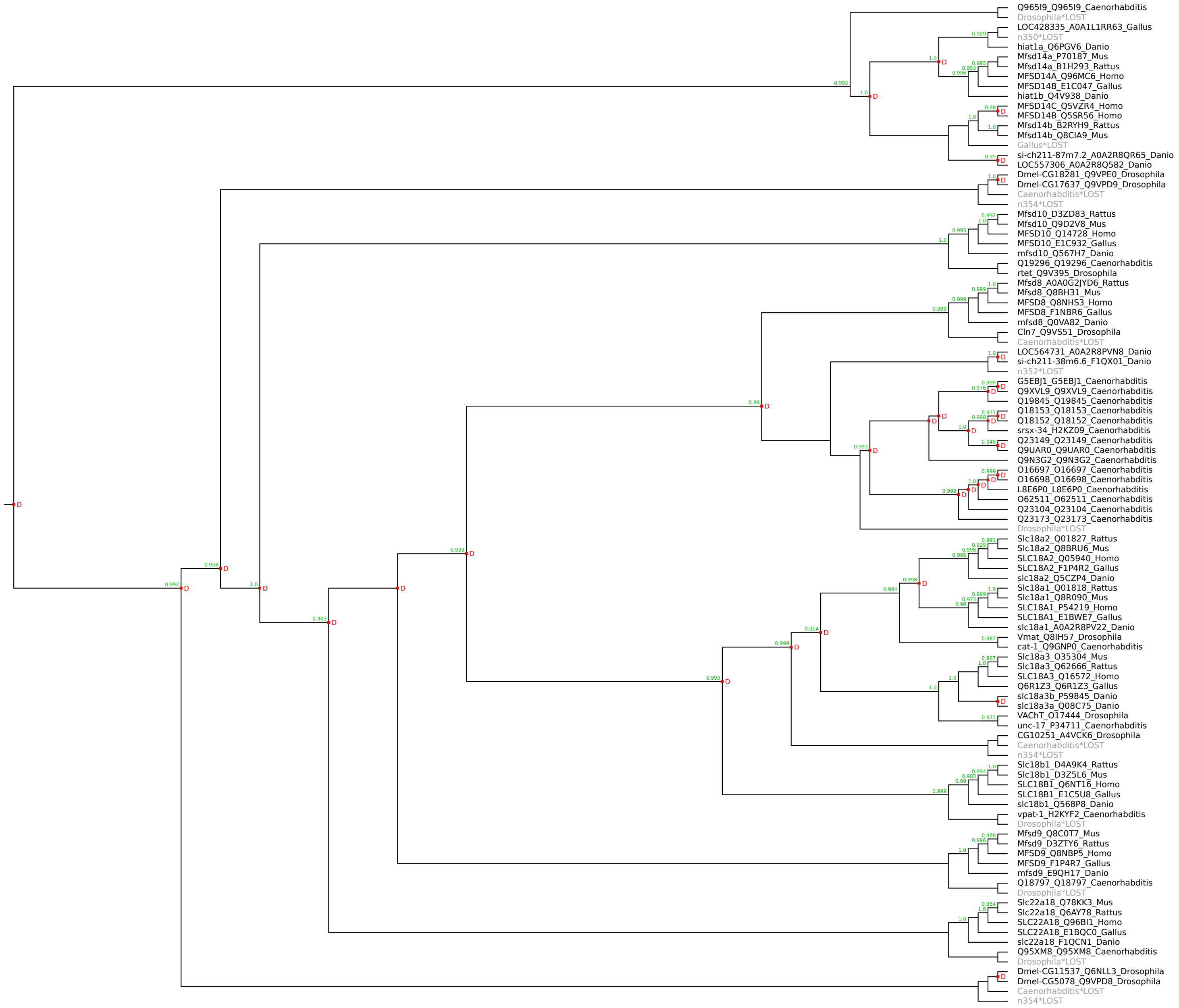

SLC19 family

Species tree:

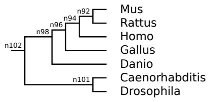

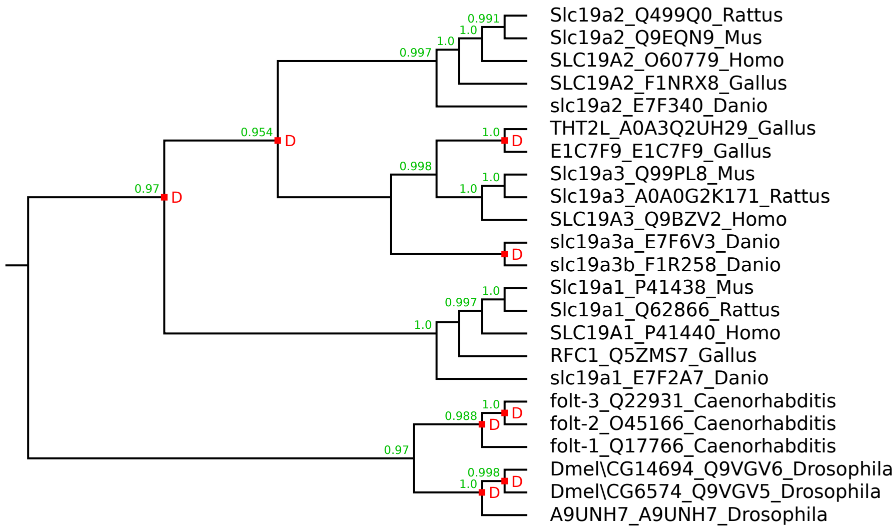

SLC20 family

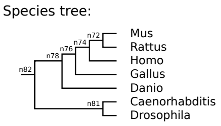

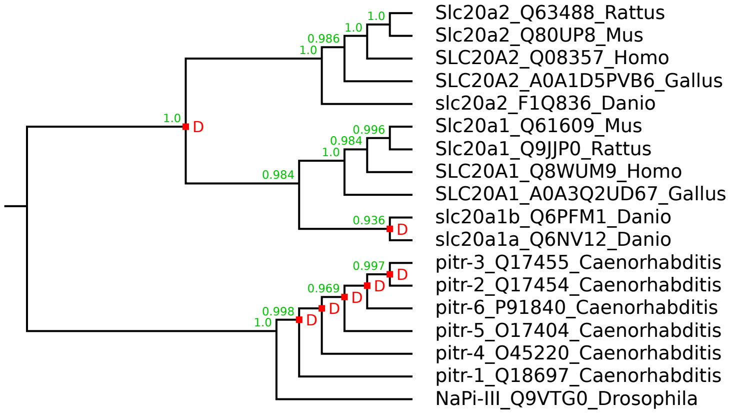

Species tree:

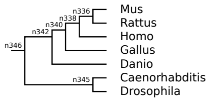

SLC21/SLCO family

Warning: this is a large figure that had to be reduced to fit on the page.
Please use the zoom function of your PDF viewer to see the details.

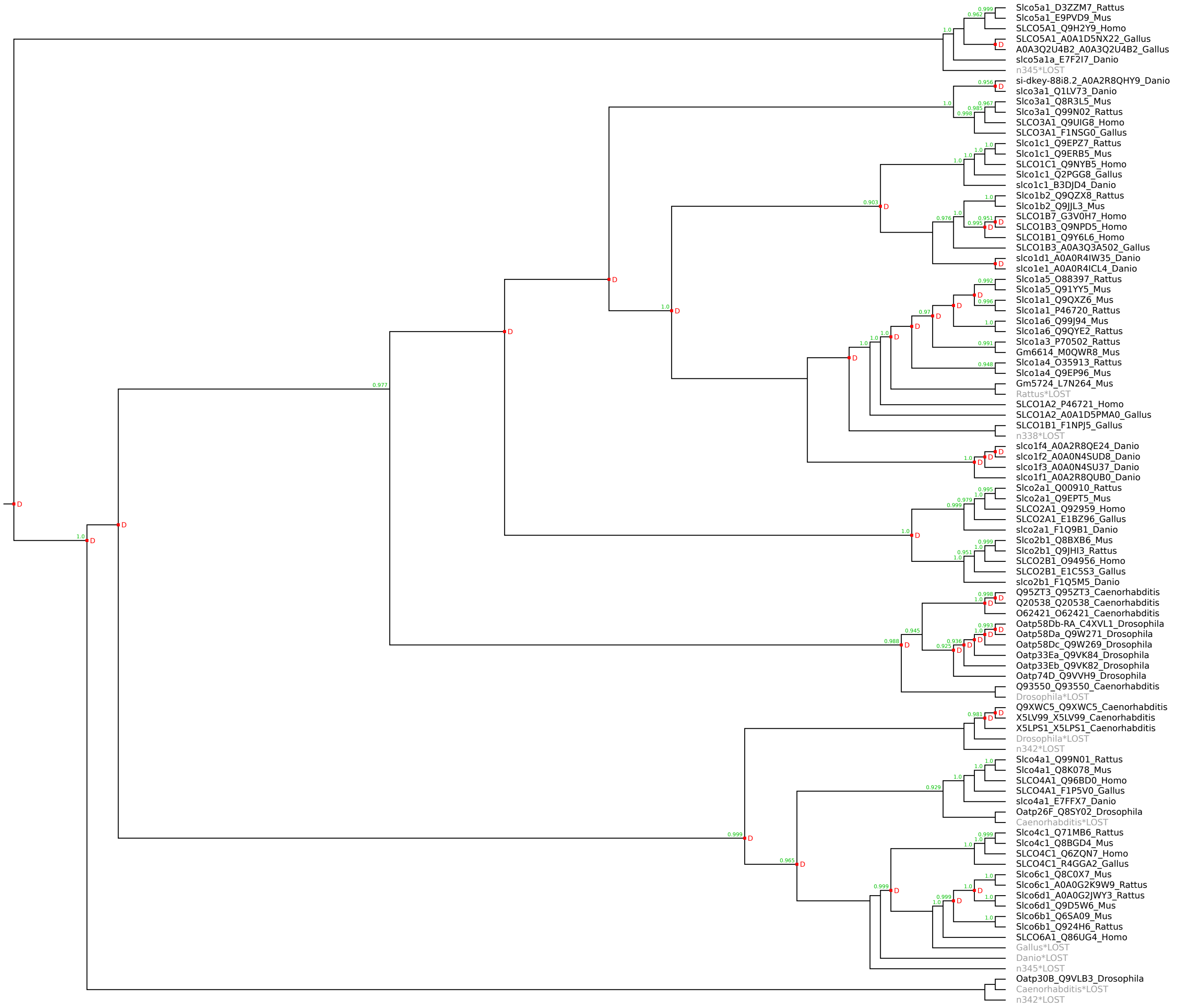

Species tree:

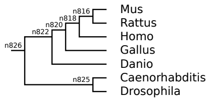

SLC22 family

Warning: this is a large figure that had to be reduced to fit on the page.
Please use the zoom function of your PDF viewer to see the details.

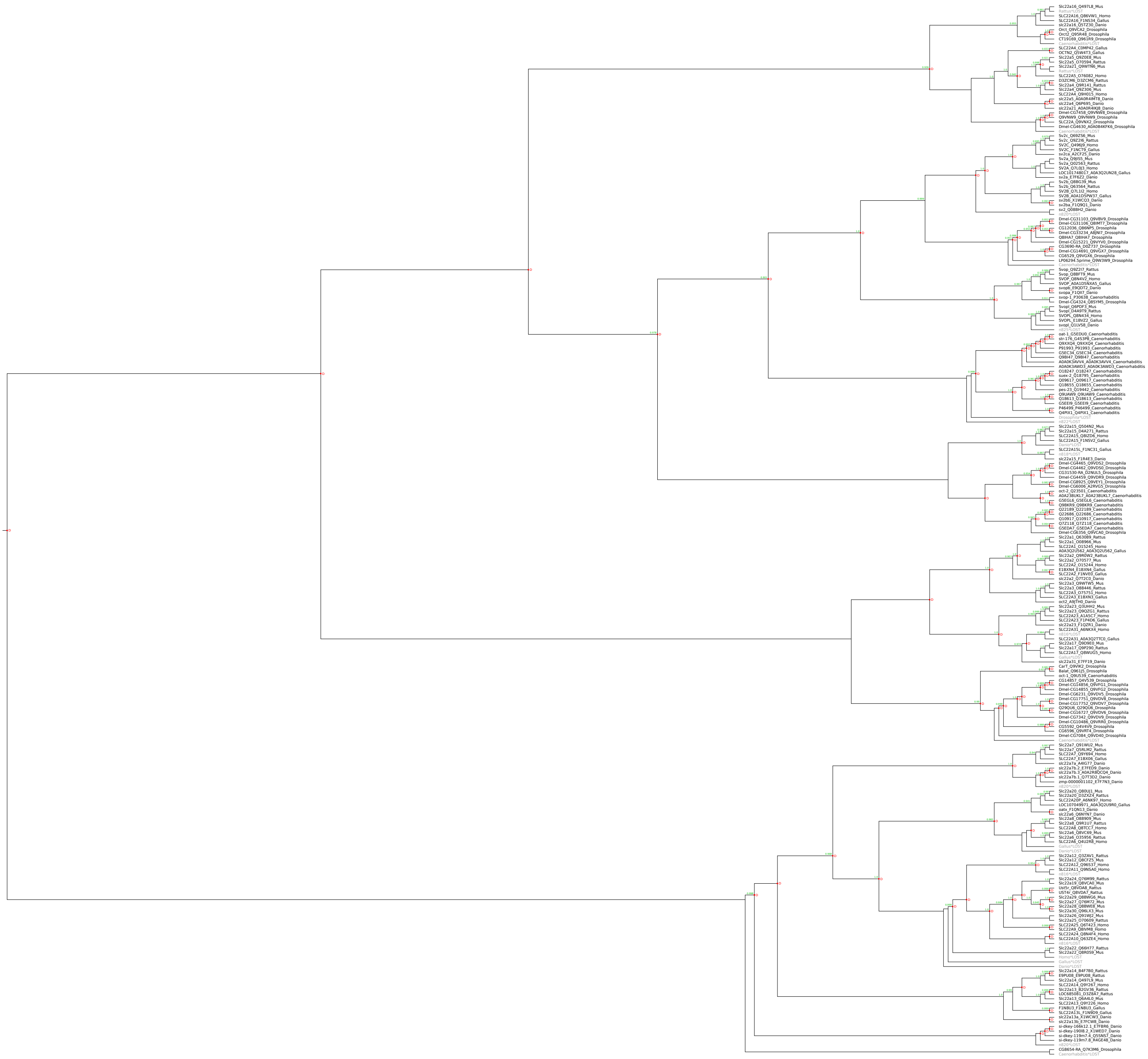

SLC23 family

Species tree:

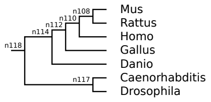

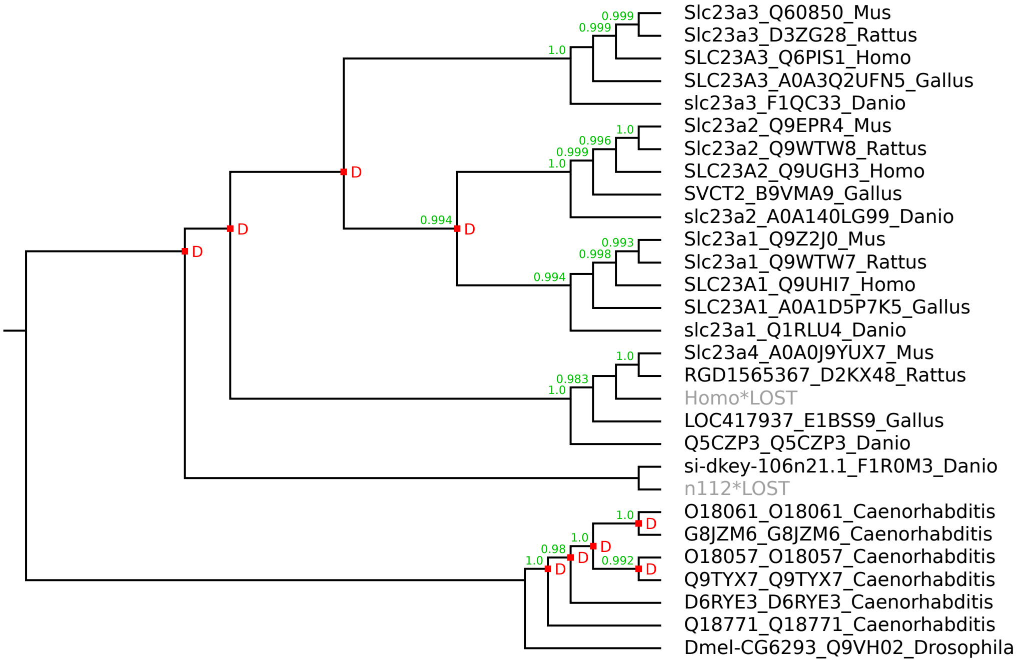

SLC24 family

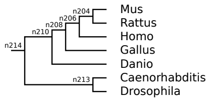

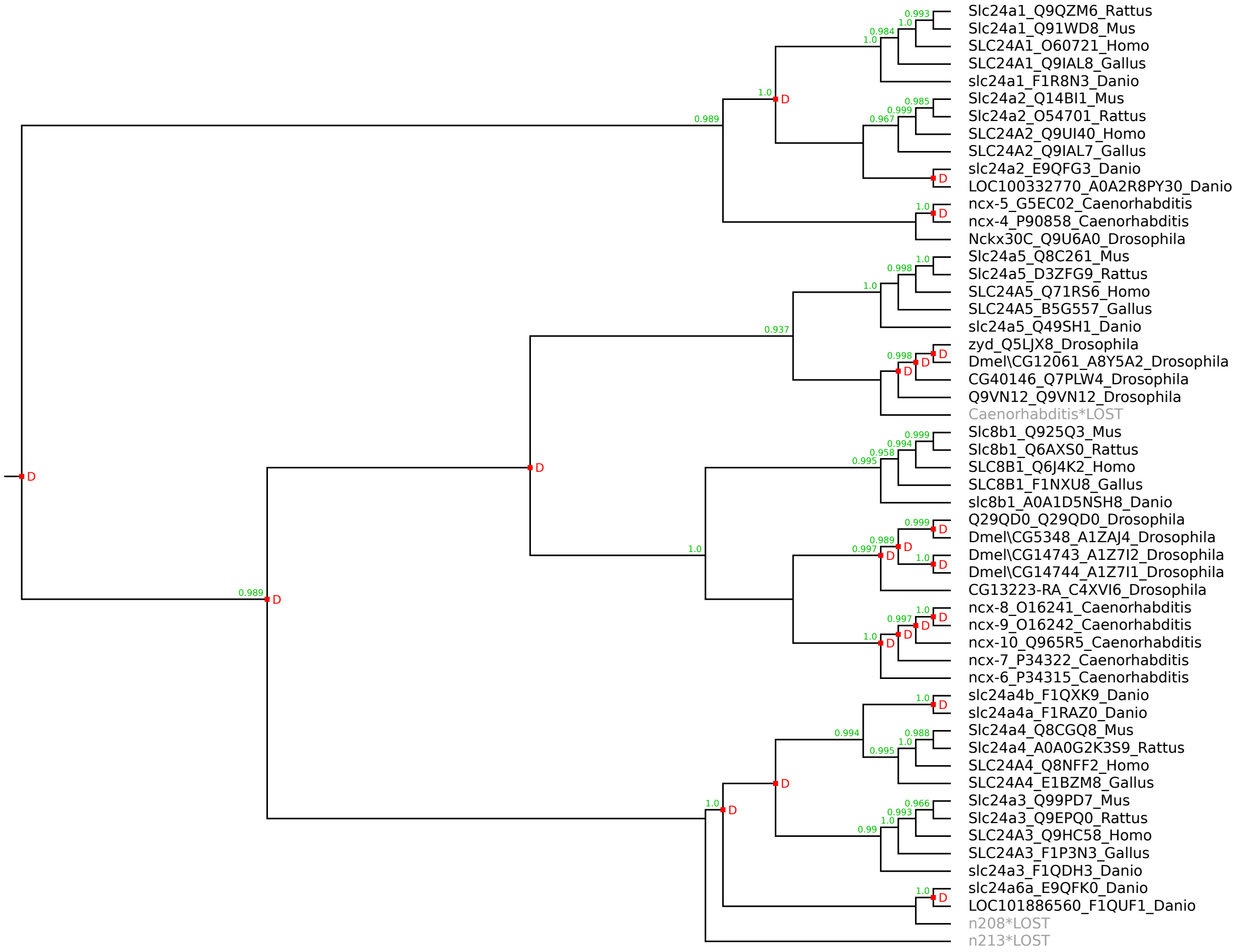

Species tree:

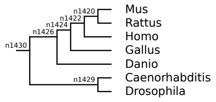

SLC25 family

Warning: this is a large figure that had to be reduced to fit on the page.
Please use the zoom function of your PDF viewer to see the details.

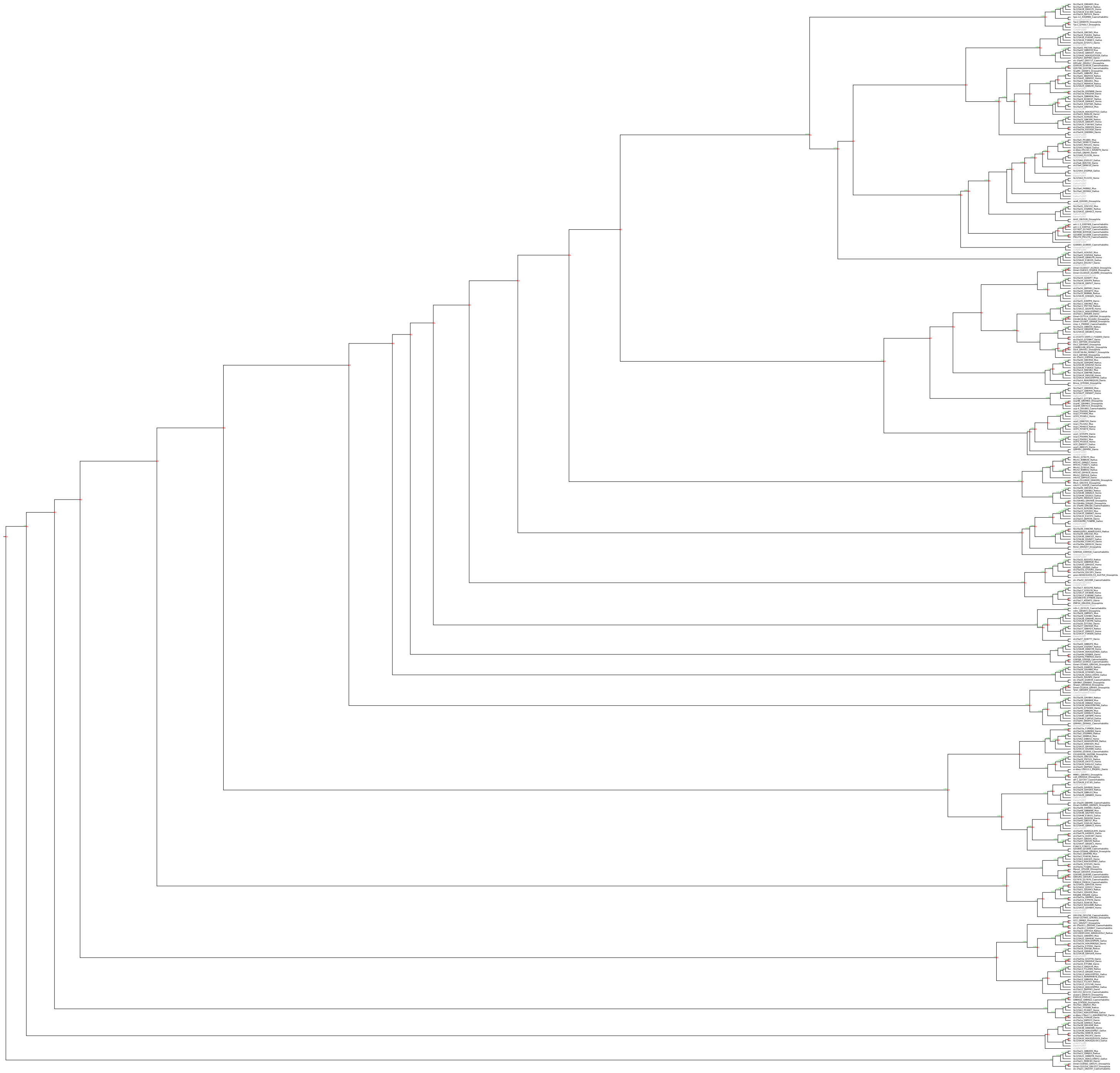

Species tree:

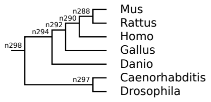

SLC26 family

Warning: this is a large figure that had to be reduced to fit on the page.
Please use the zoom function of your PDF viewer to see the details.

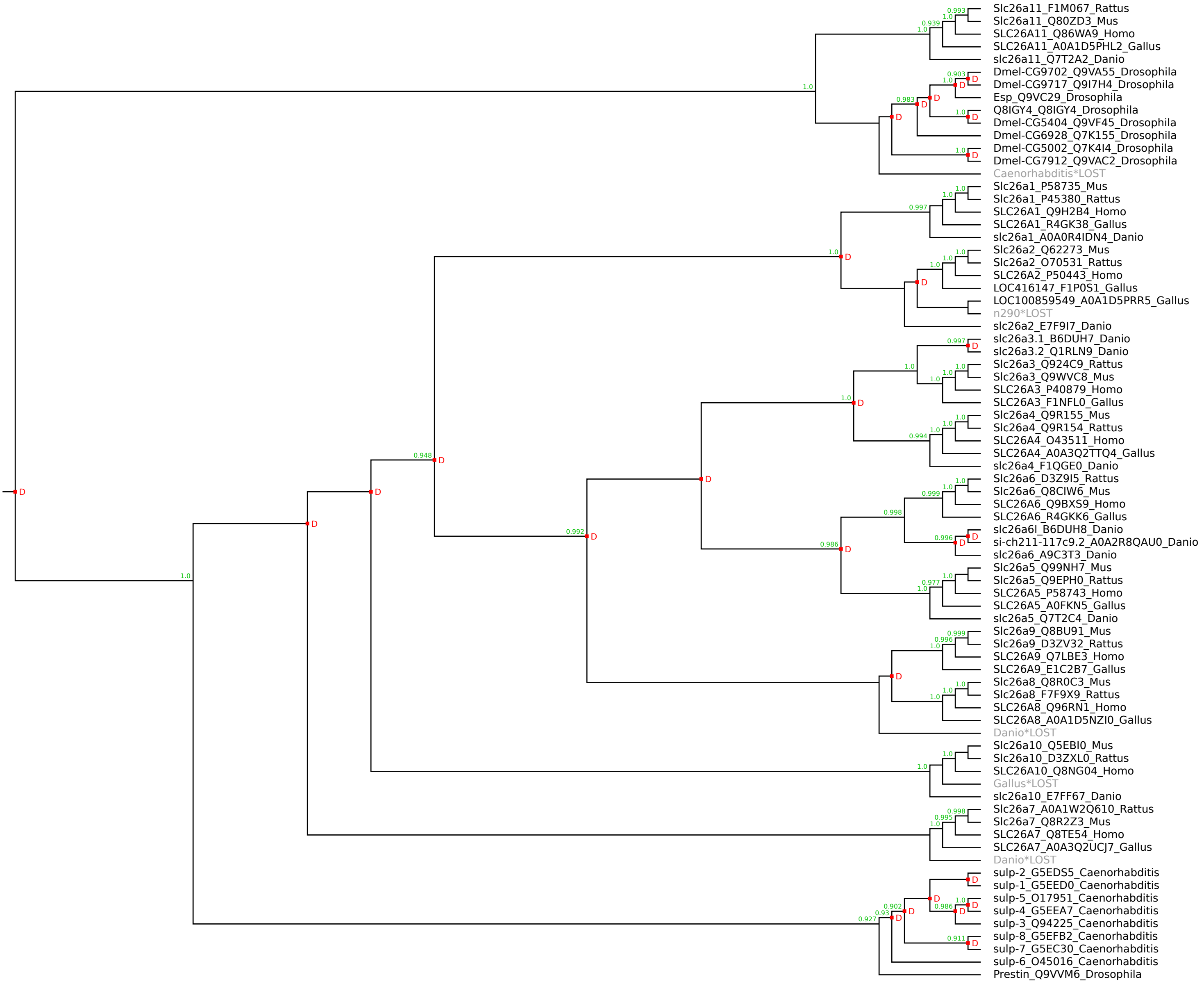

SLC28 family

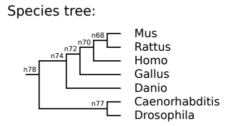

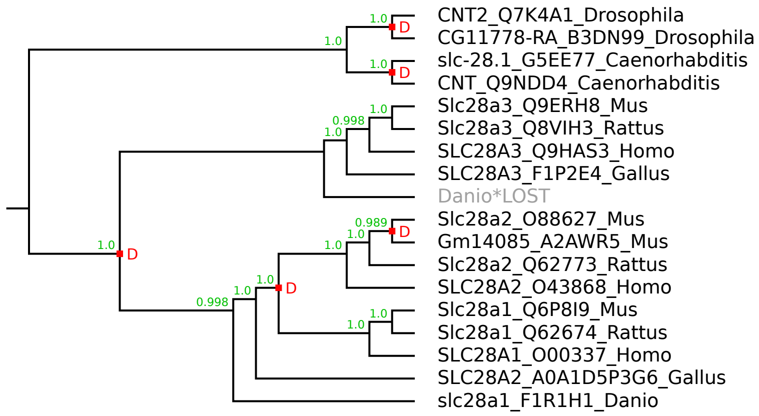

SLC29 family

Species tree:

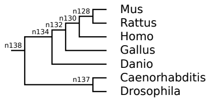

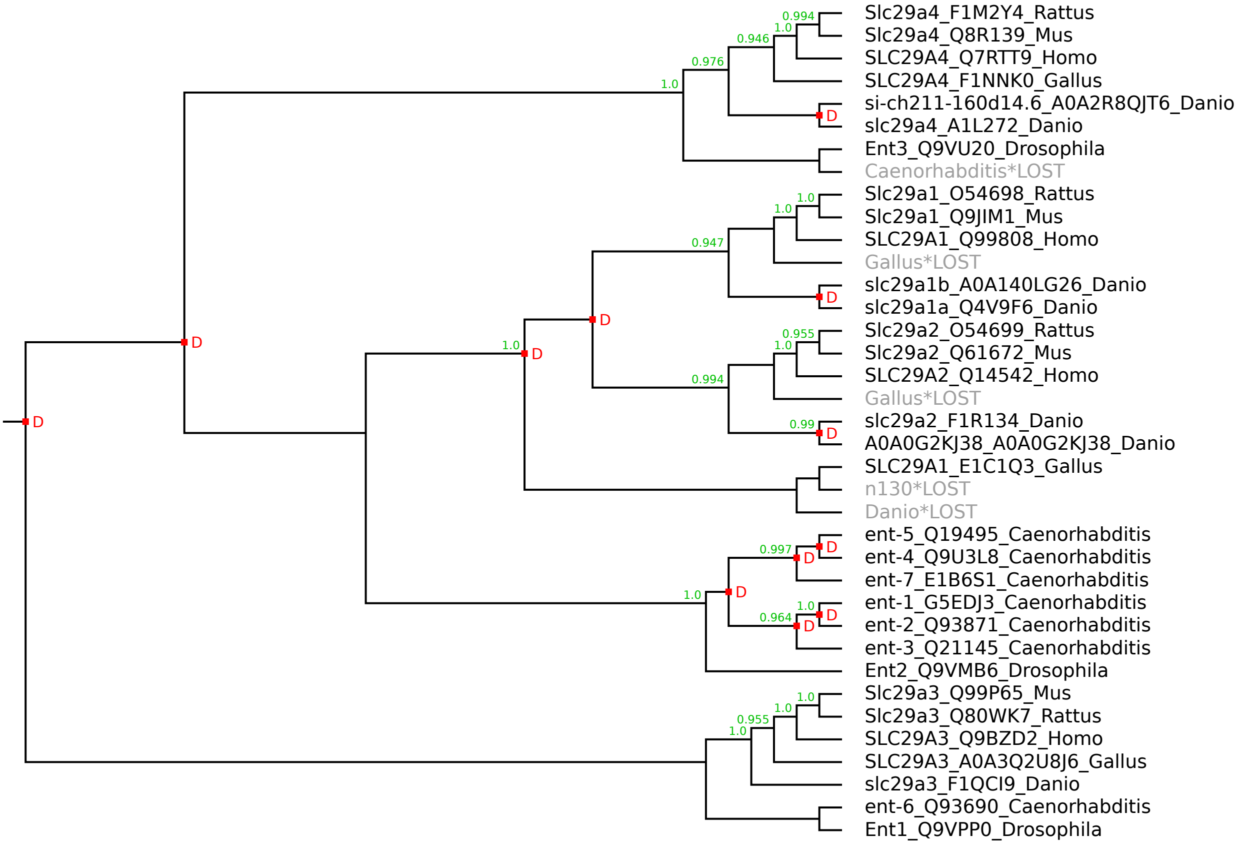

SLC30 family

Species tree:

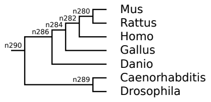

Warning: this is a large figure that had to be reduced to fit on the page.
Please use the zoom function of your PDF viewer to see the details.

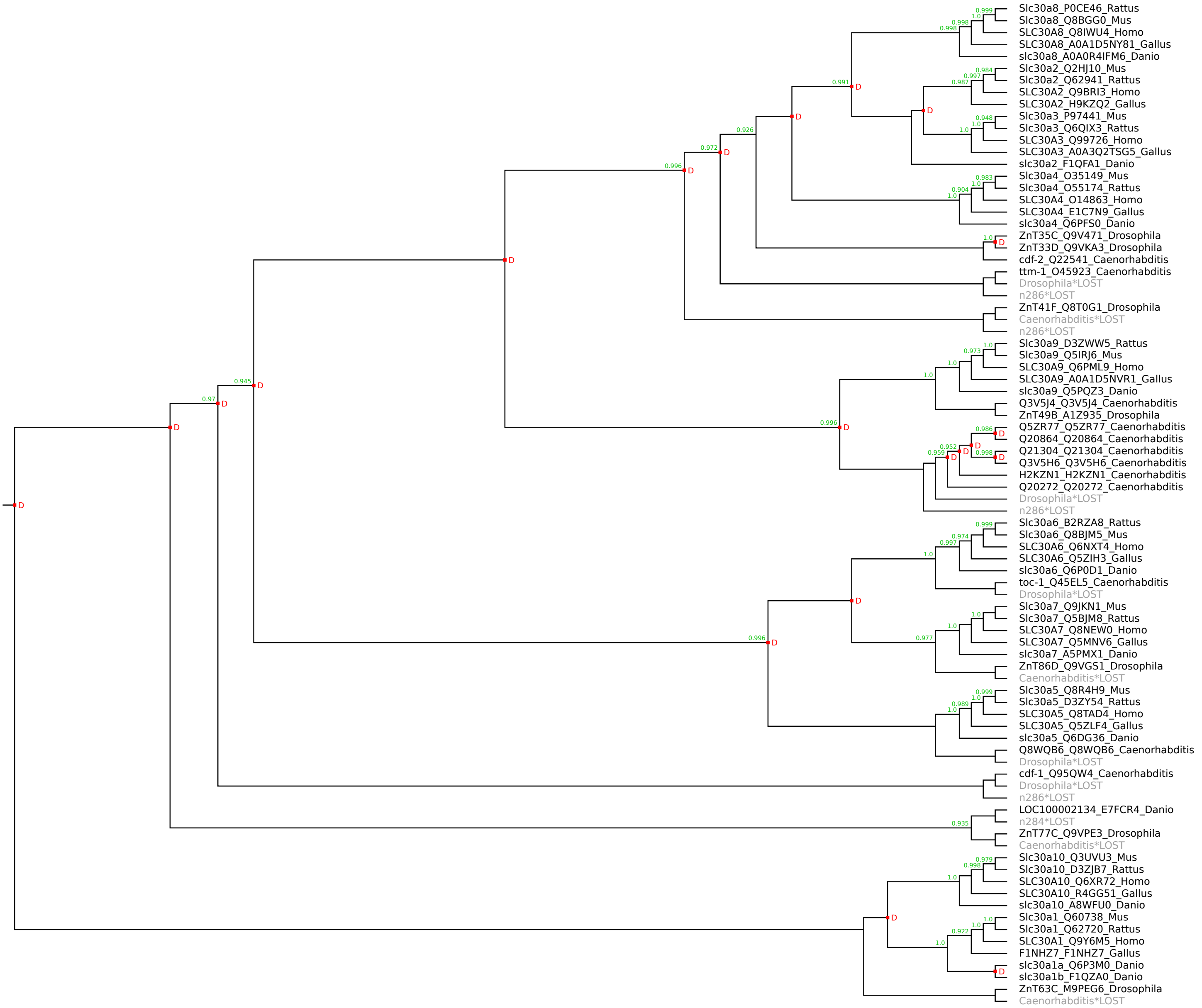

SLC31 family

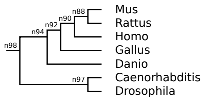

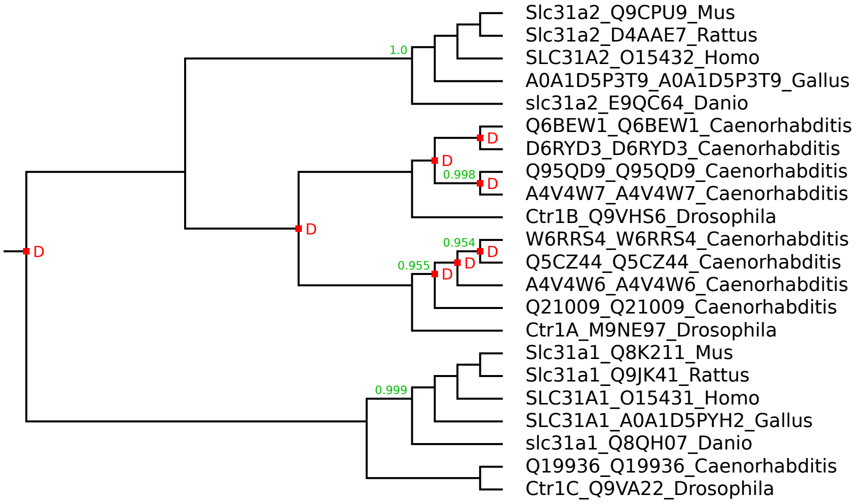

SLC32 family

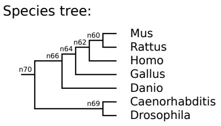

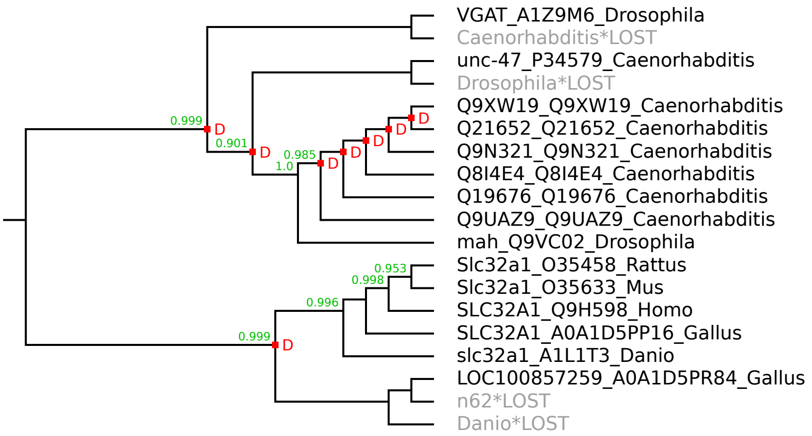

SLC33 family

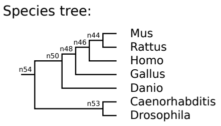

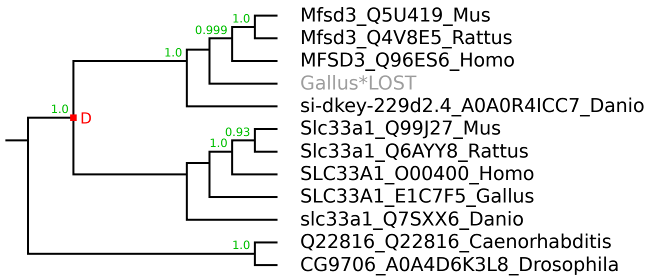

Species tree:

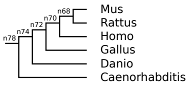

SLC34 family

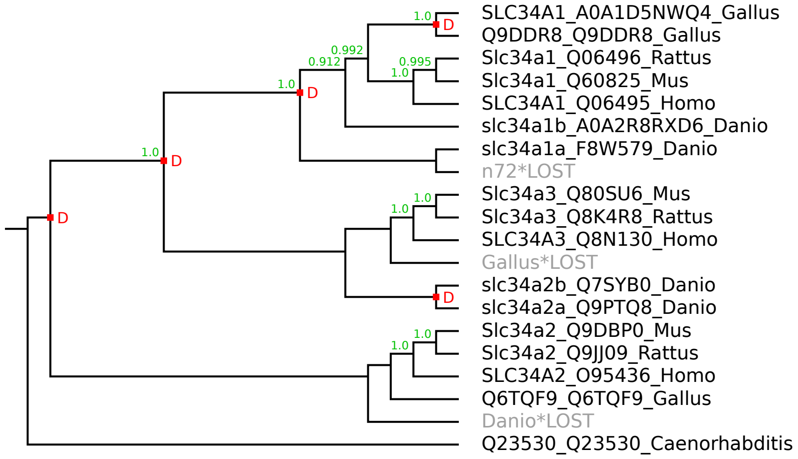

Species tree:

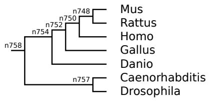

SLC35 family

Warning: this is a large figure that had to be reduced to fit on the page.
Please use the zoom function of your PDF viewer to see the details.

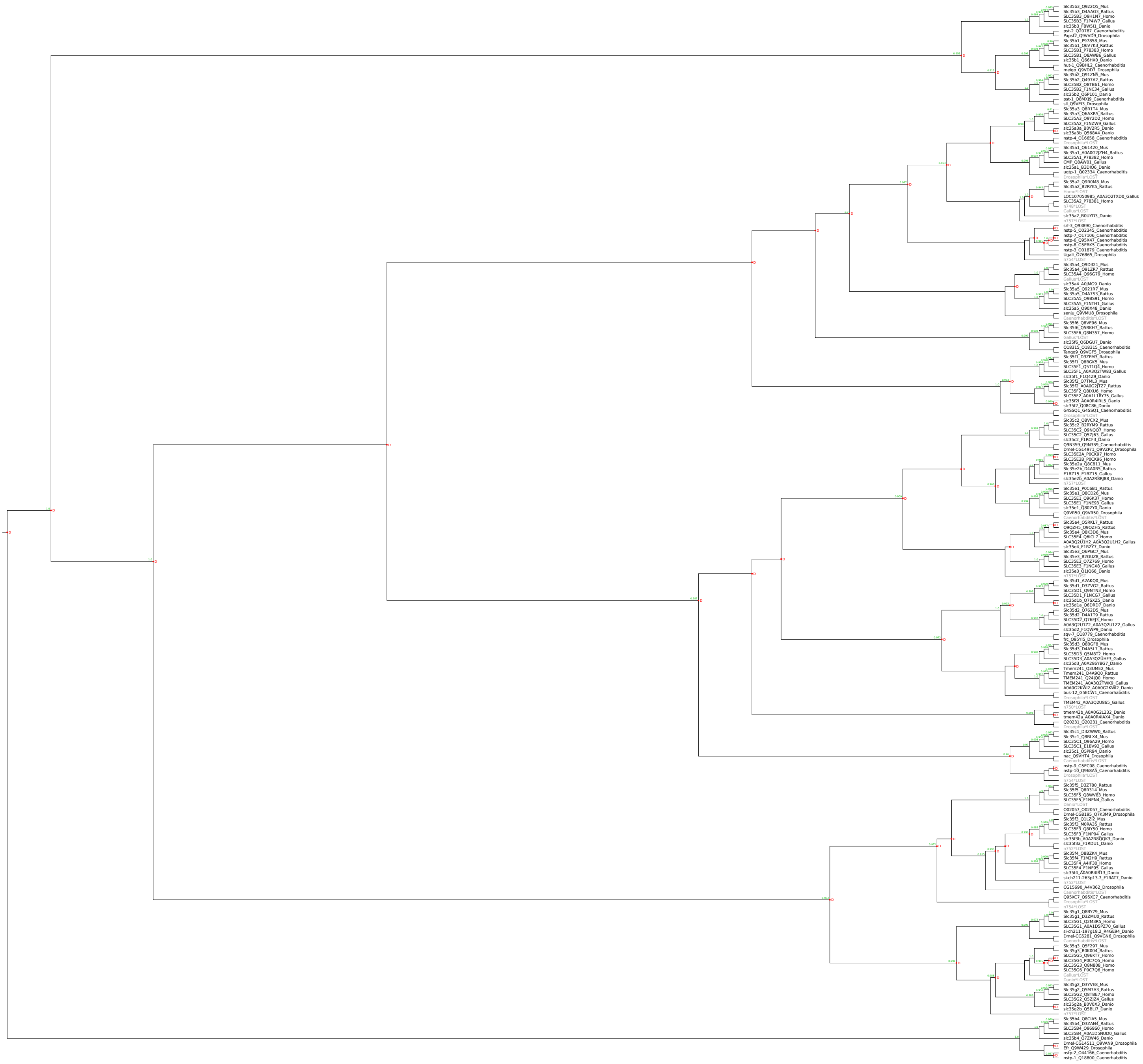

SLC36 family

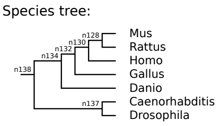

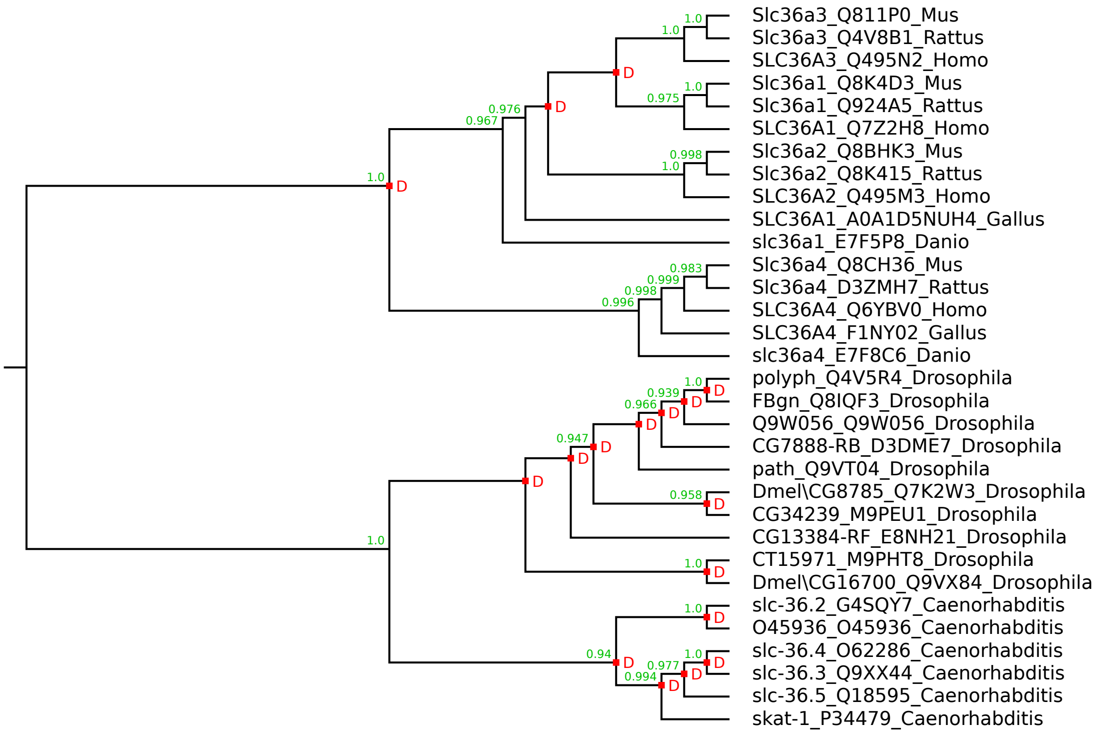

SLC37 family

Species tree:

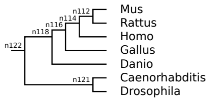

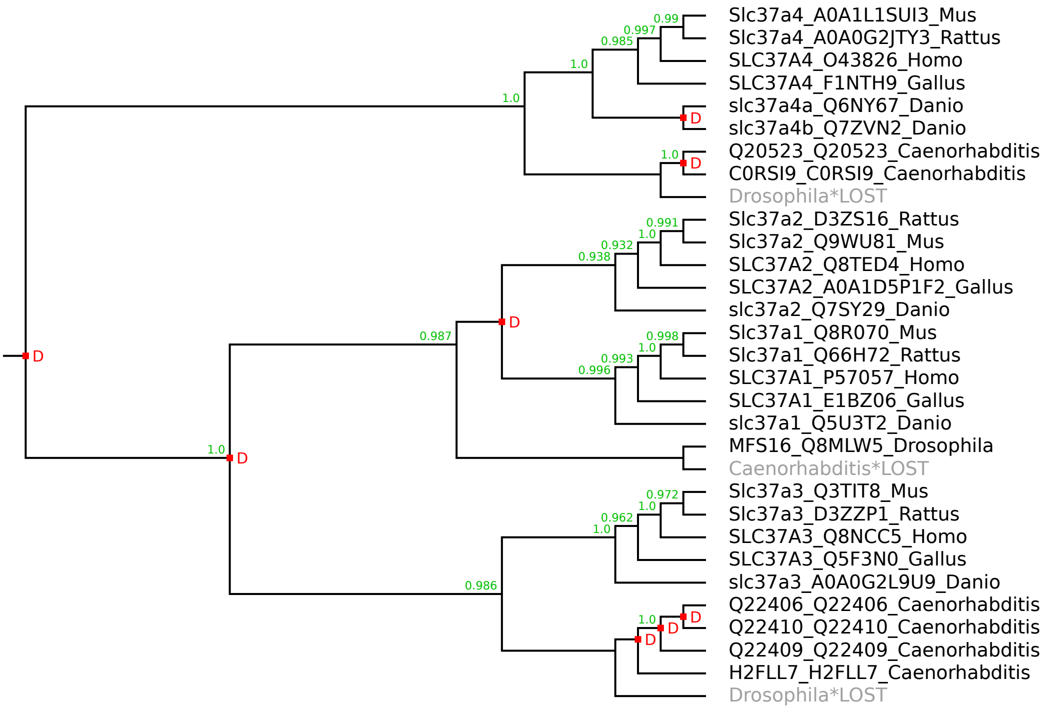

SLC38 family

Species tree:

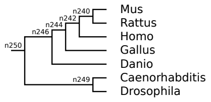

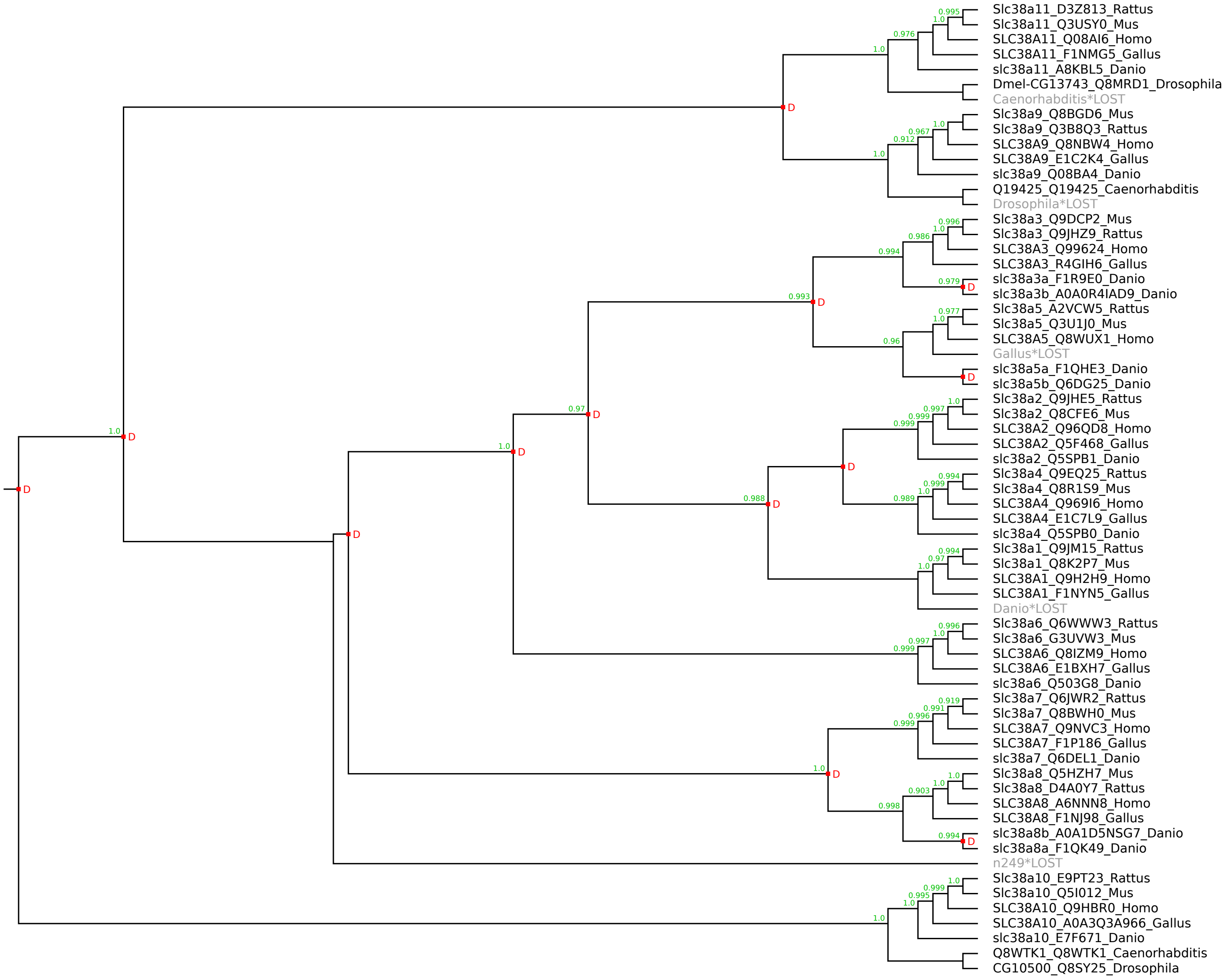

SLC39 family

Species tree:

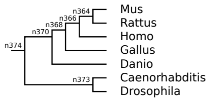

Warning: this is a large figure that had to be reduced to fit on the page.
Please use the zoom function of your PDF viewer to see the details.

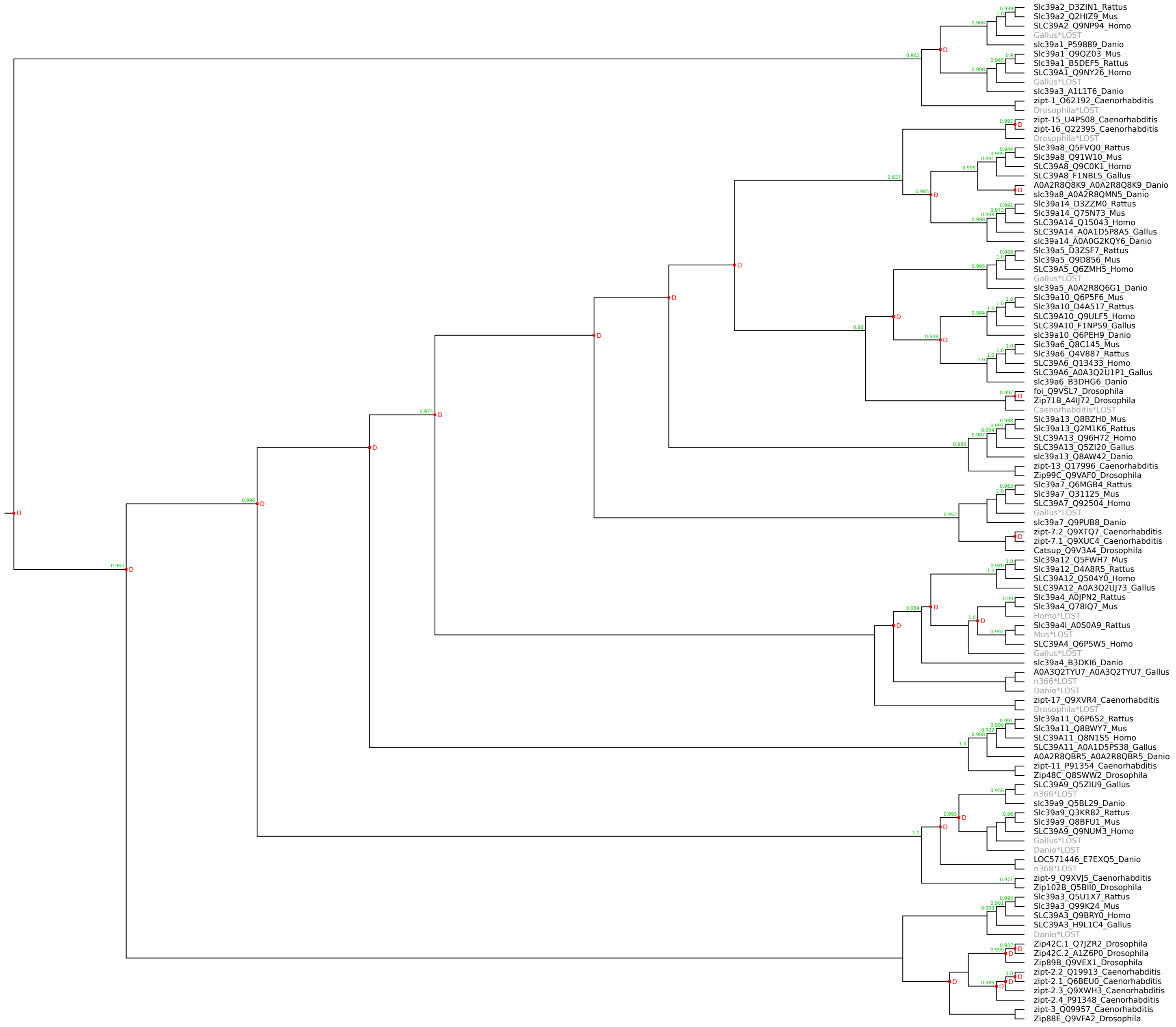

SLC40 family

Species tree:

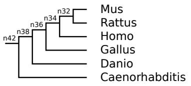

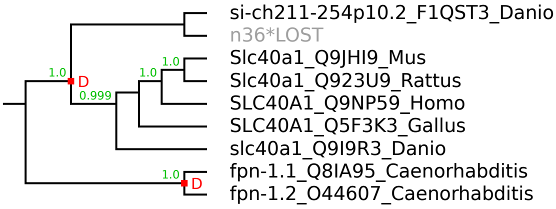

SLC41 family

Species tree:

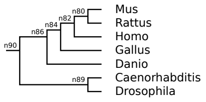

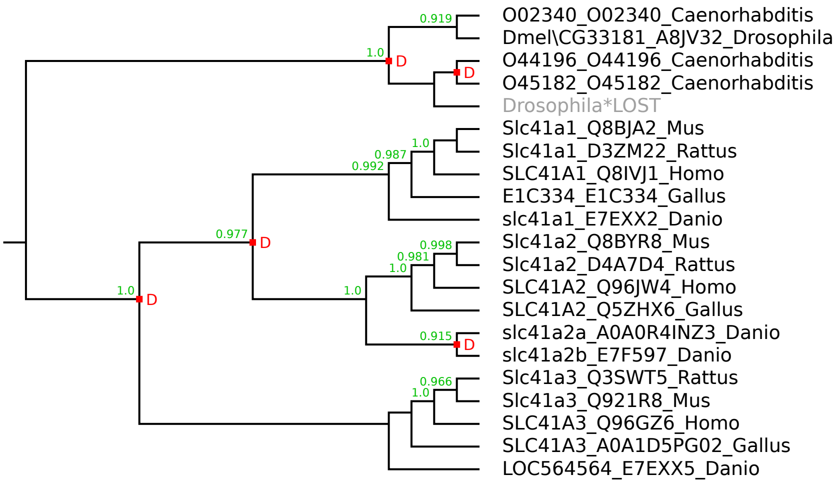

SLC42 family

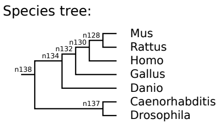

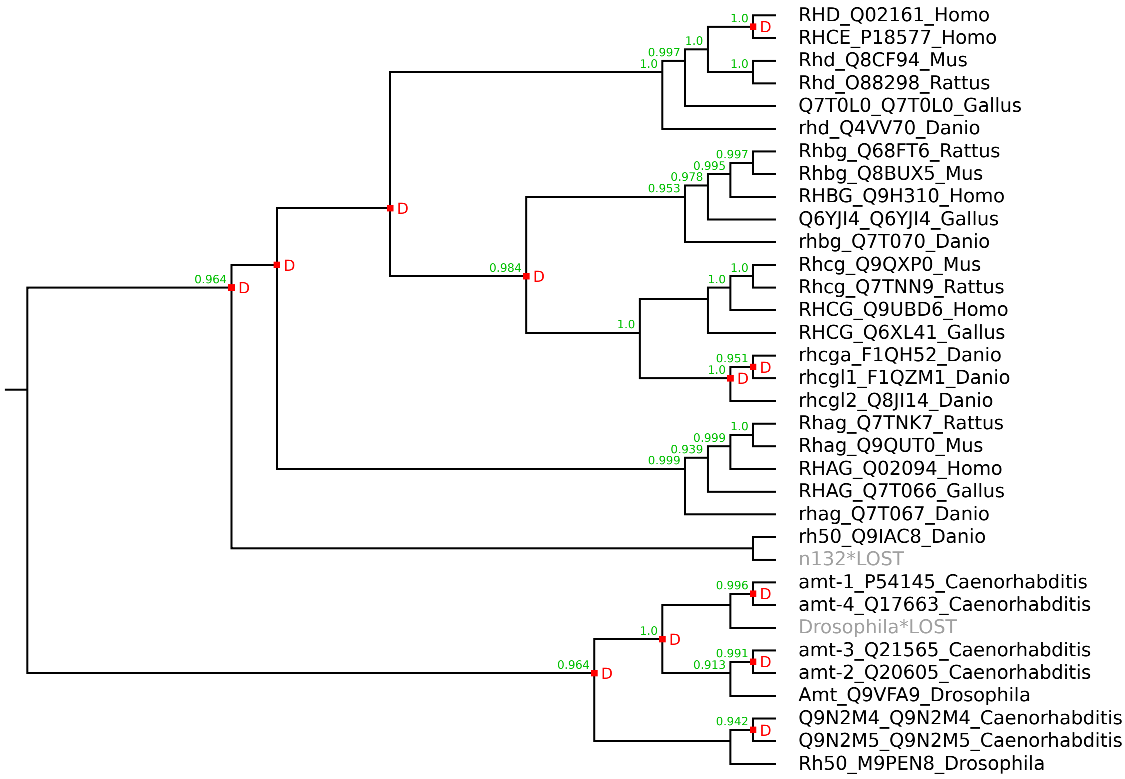

SLC43 family

Species tree:

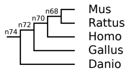

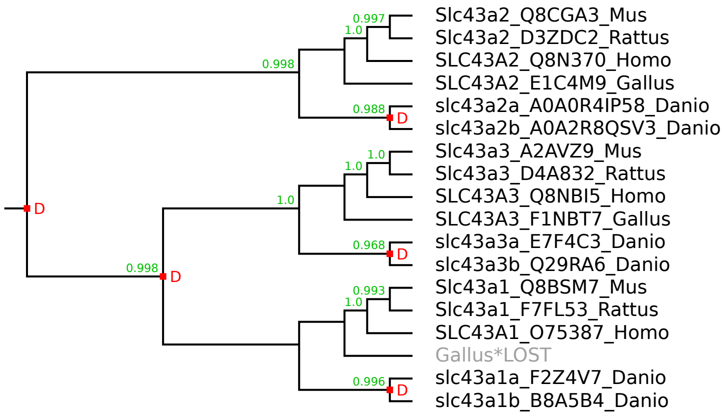

SLC44 family

Species tree:

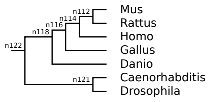

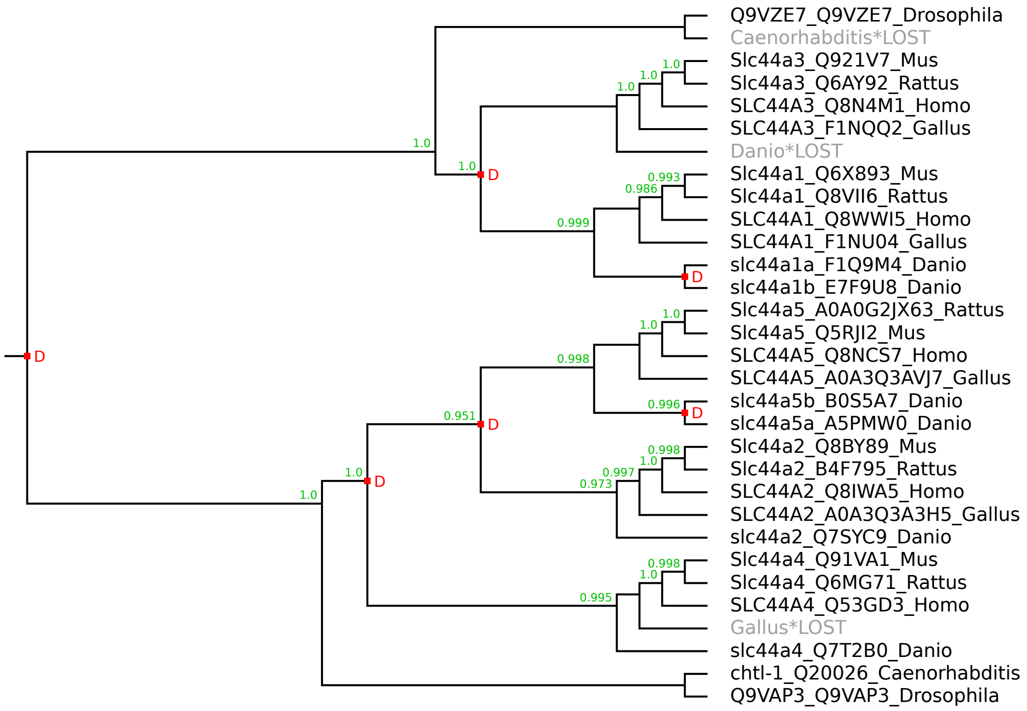

SLC45 family

Species tree:

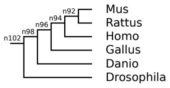

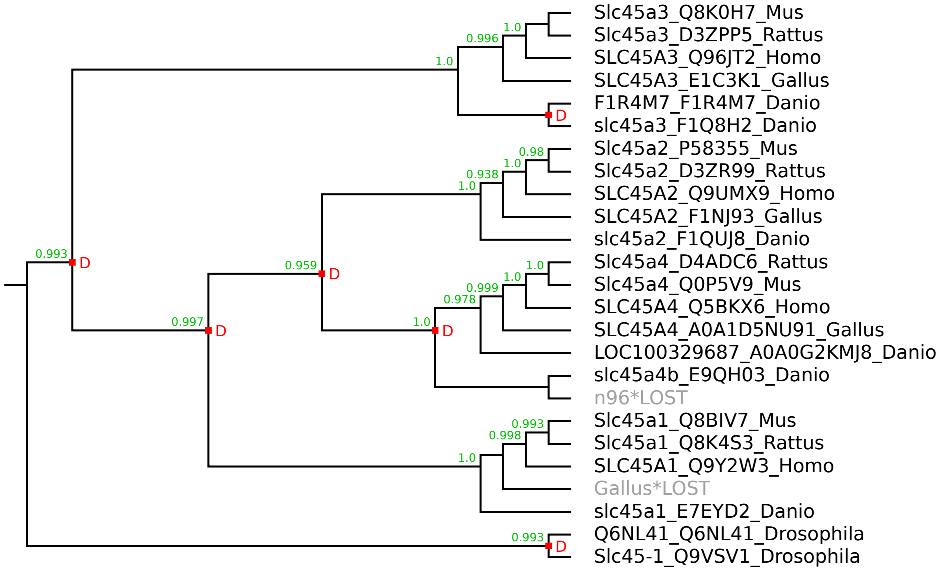

SLC46 family

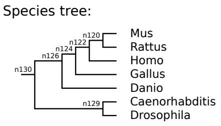

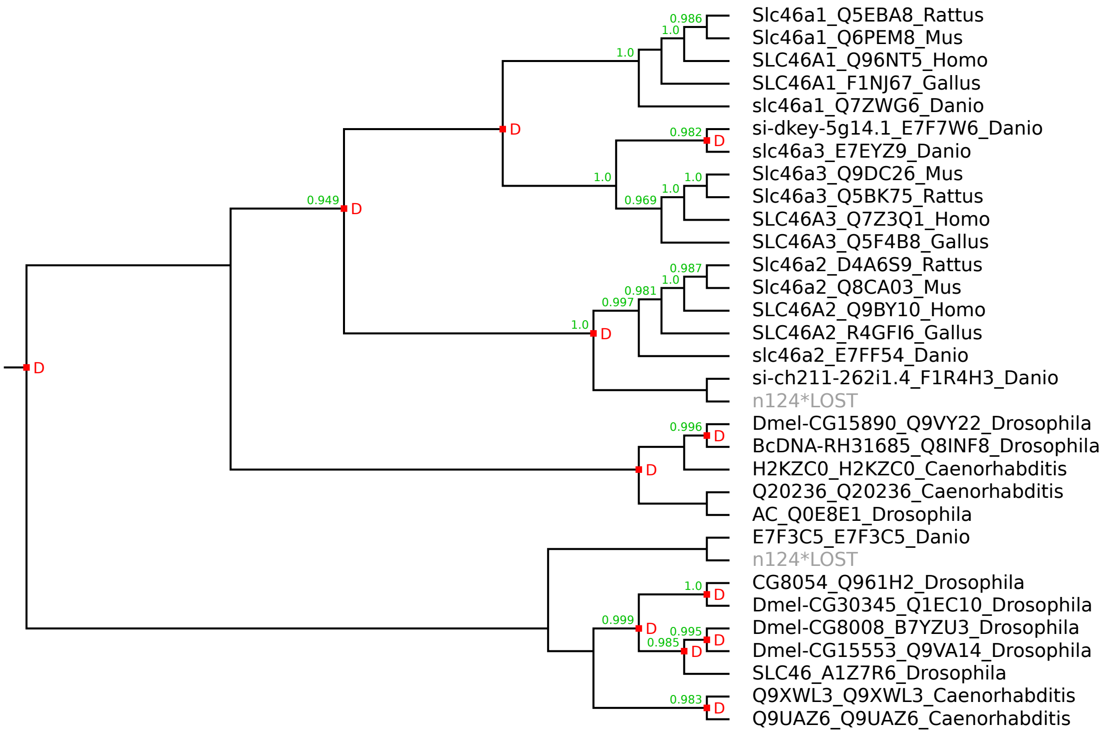

SLC47 family

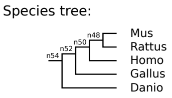

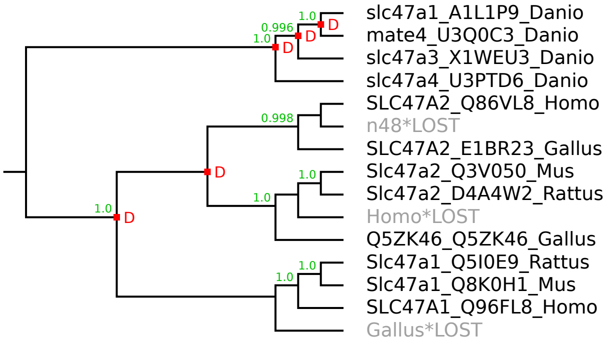

SLC48 family

Species tree:

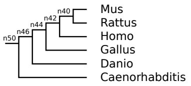

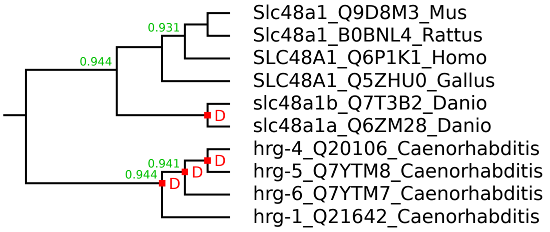

SLC49 family

Species tree:

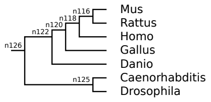

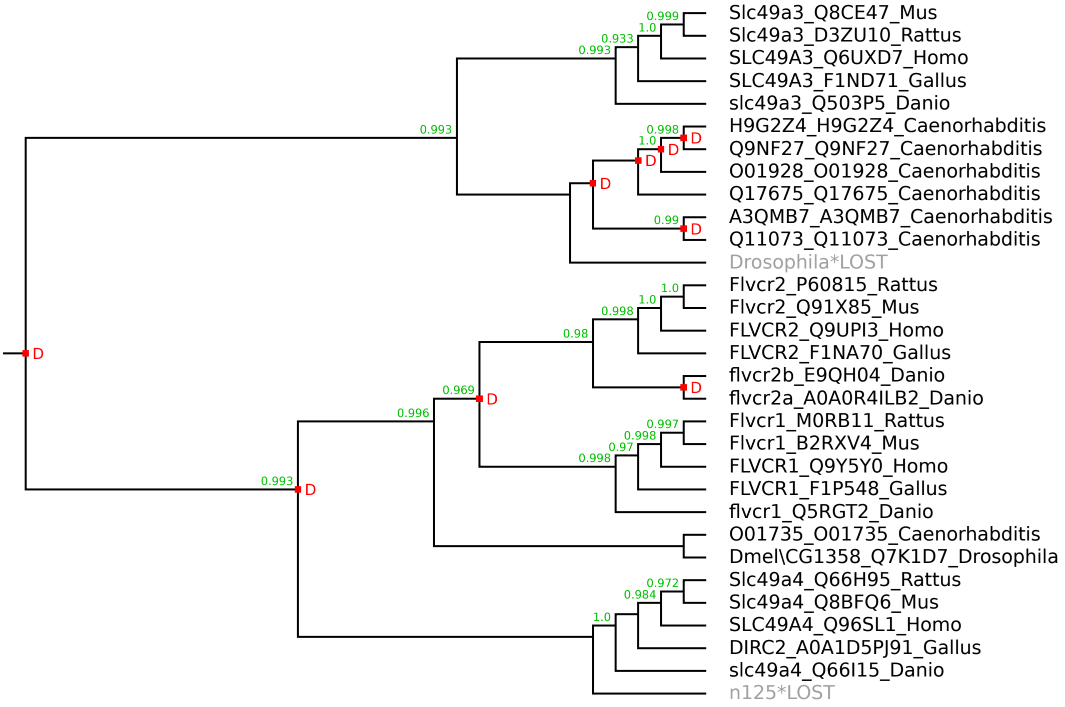

SLC50 family

Species tree:

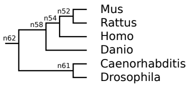

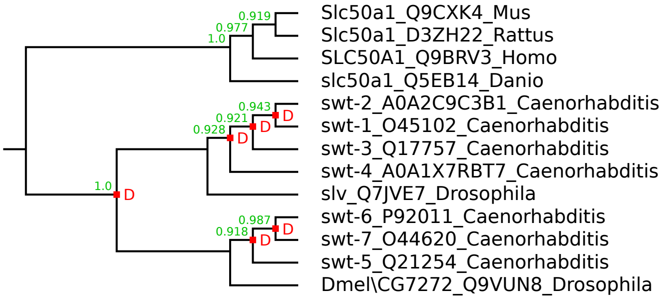

SLC51A family

Species tree:

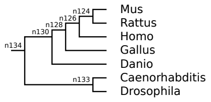

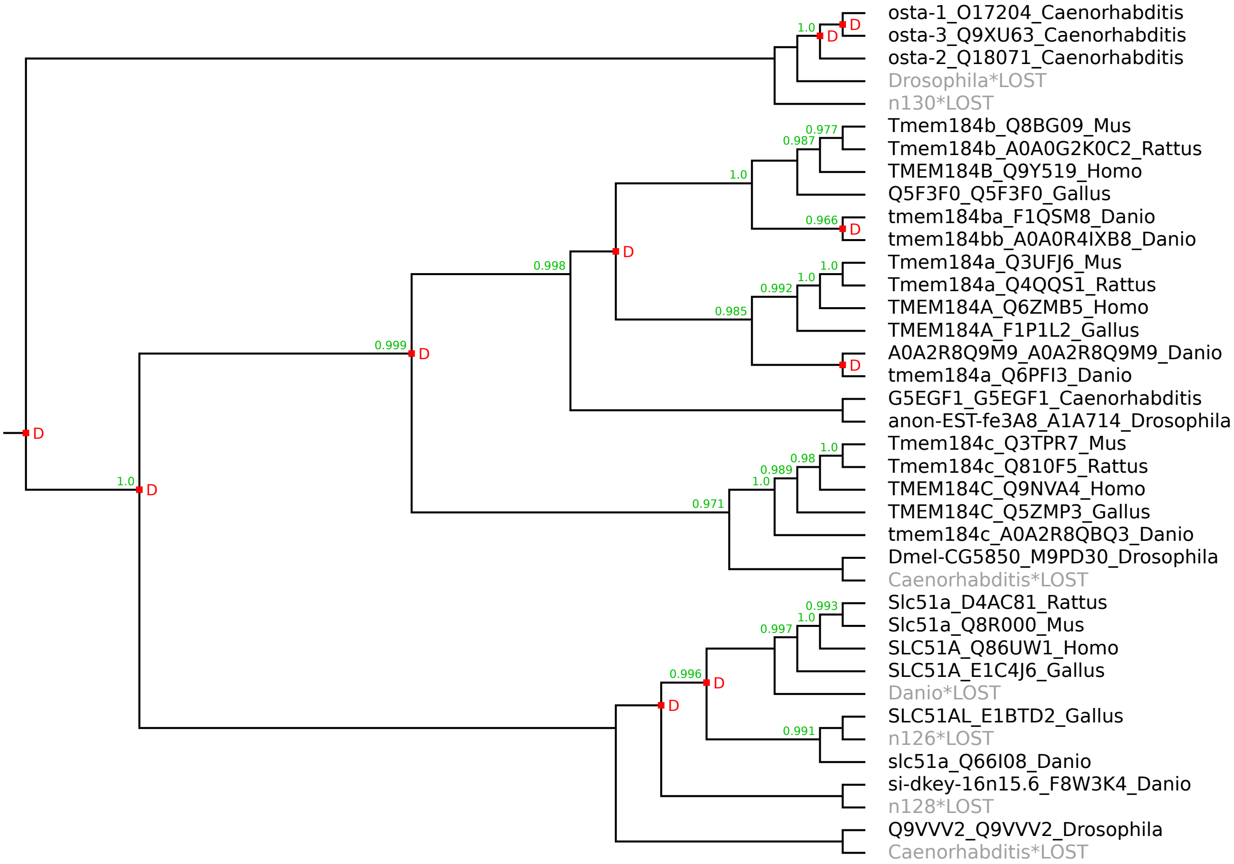

Species tree:

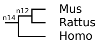

SLC51B family

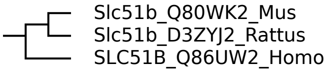

Species tree:

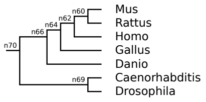

SLC52 family

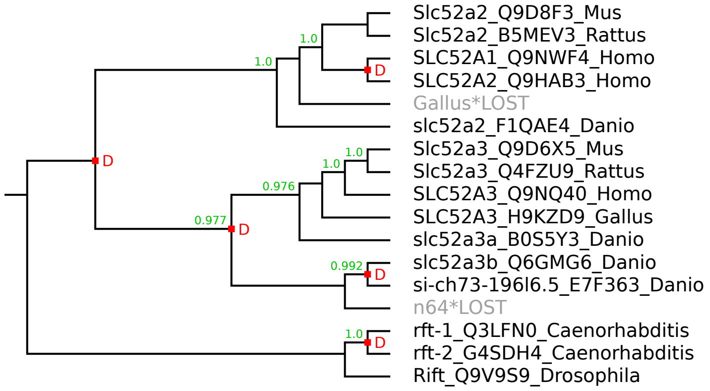

SLC53 family

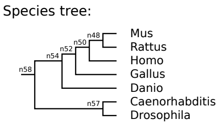

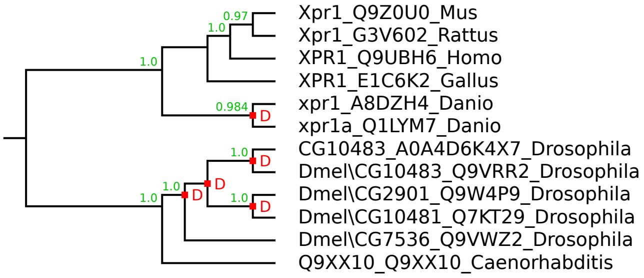

SLC54 family

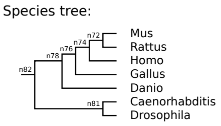

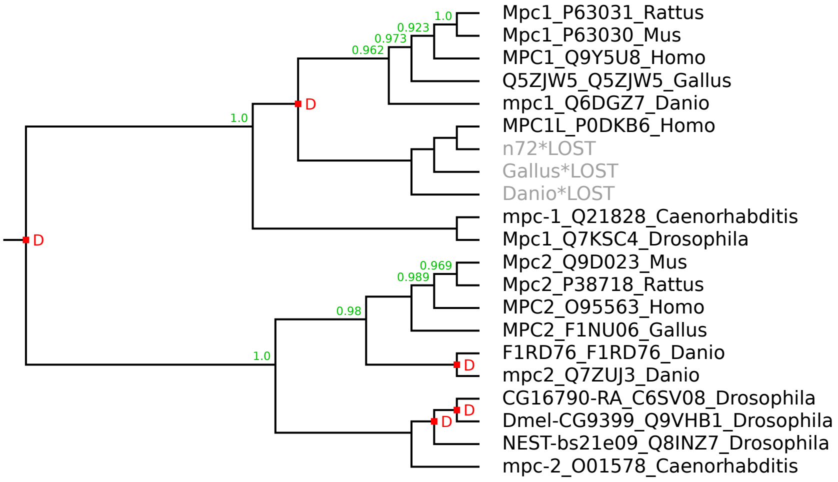

SLC55 family

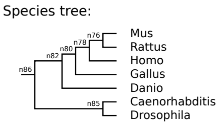

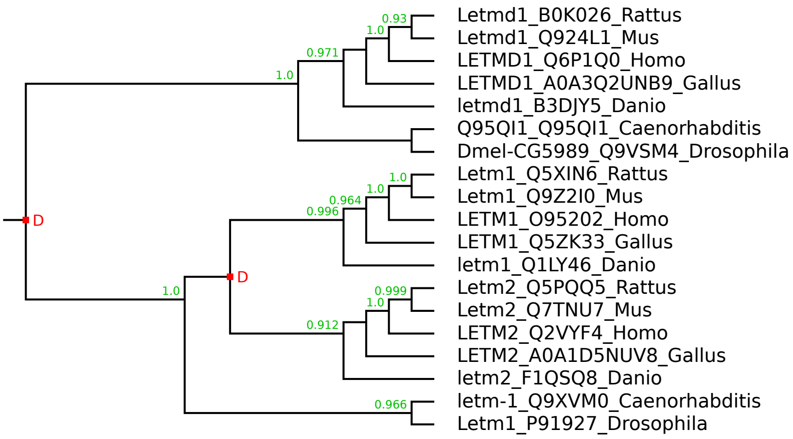

SLC56 family

Species tree:

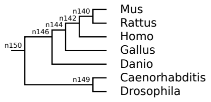

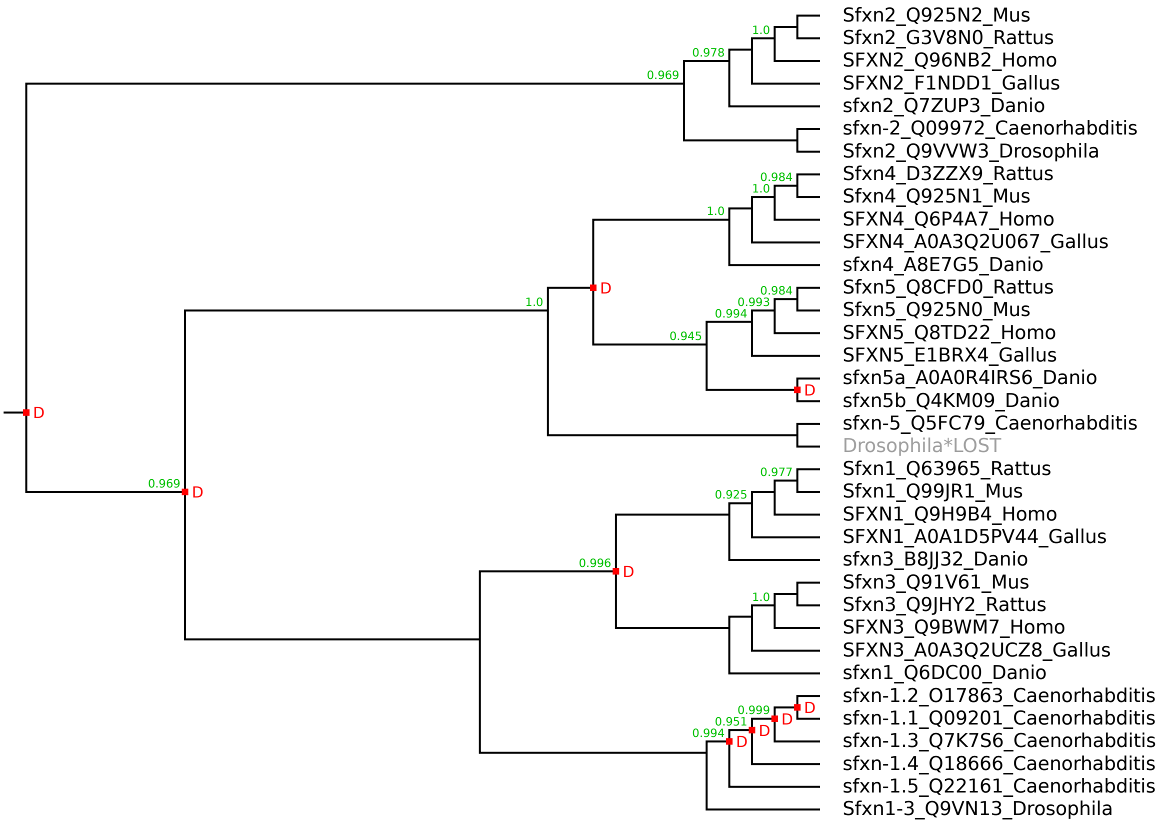

SLC57 family

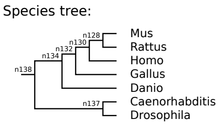

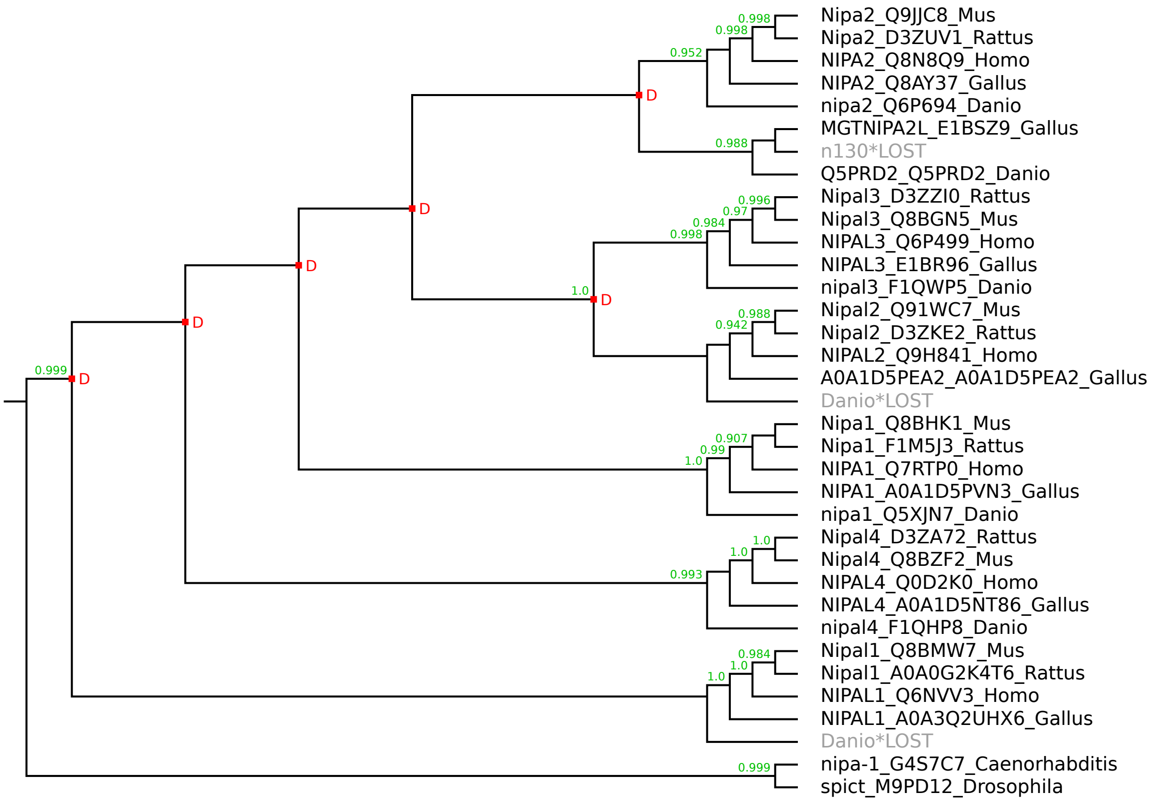

SLC58 family

Species tree:

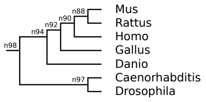

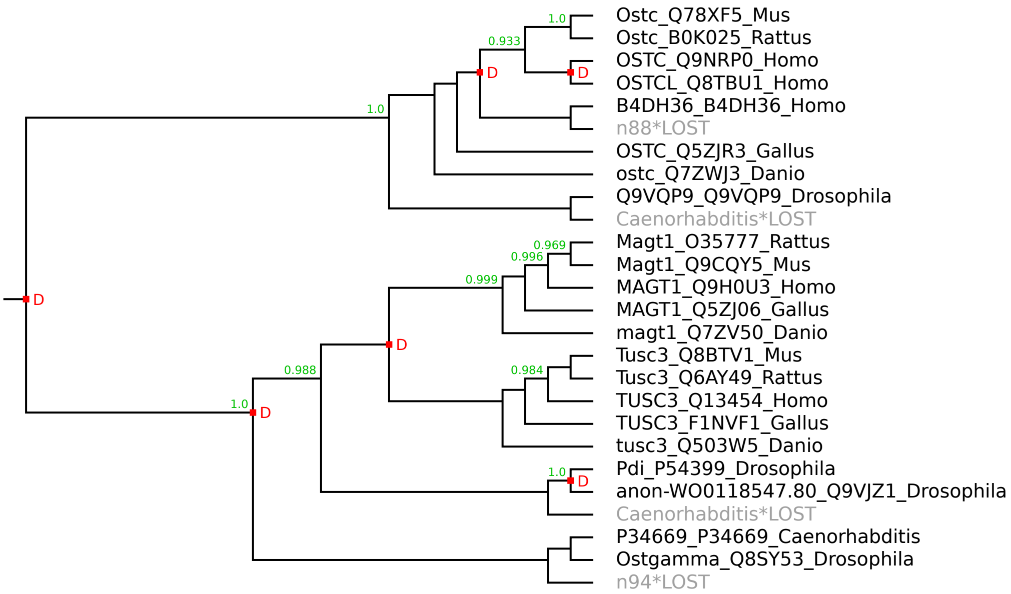

SLC59 family

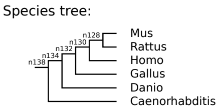

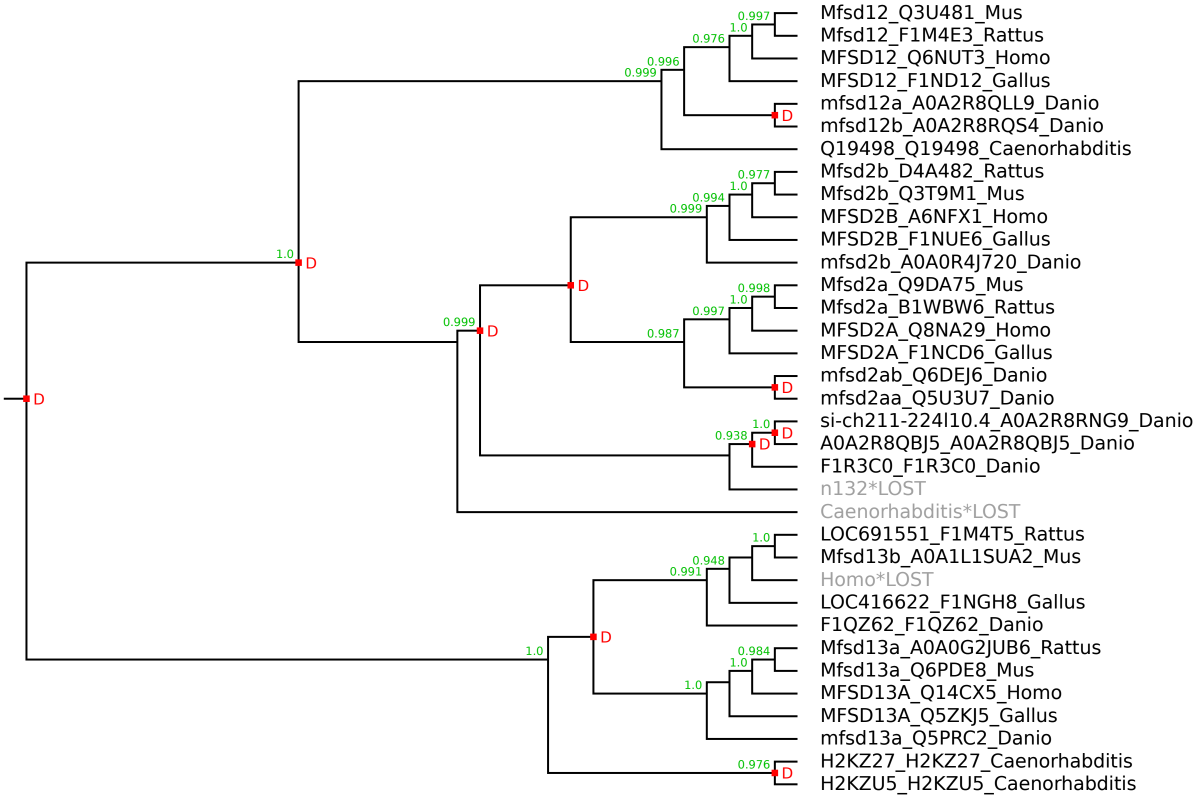

SLC60 family

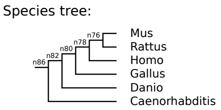

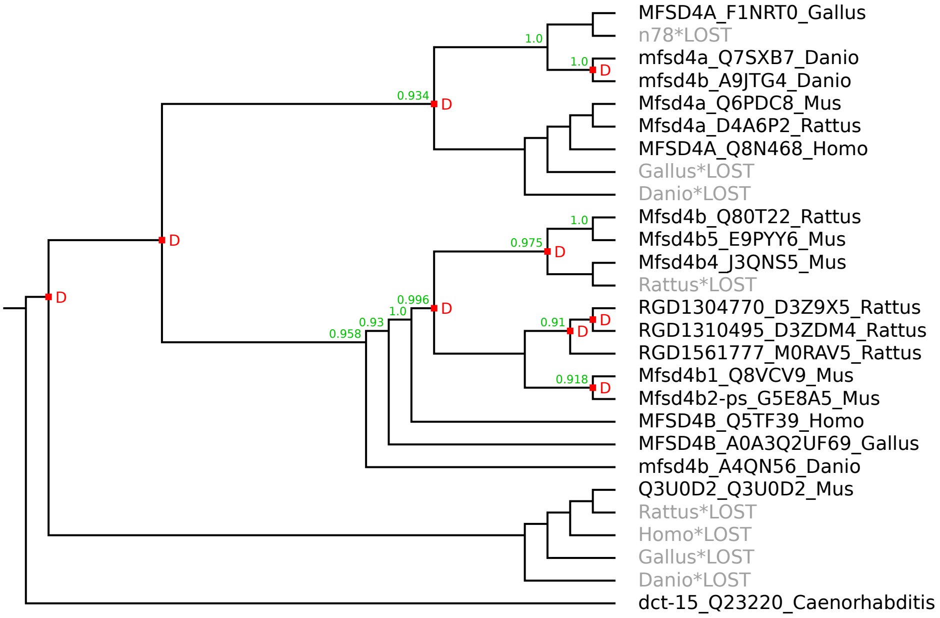

SLC61 family

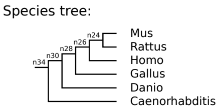

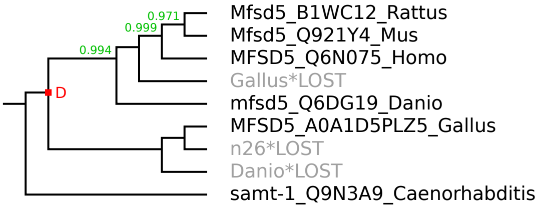

SLC62 family

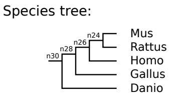

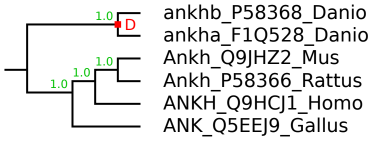

SLC63 family

Species tree:

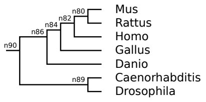

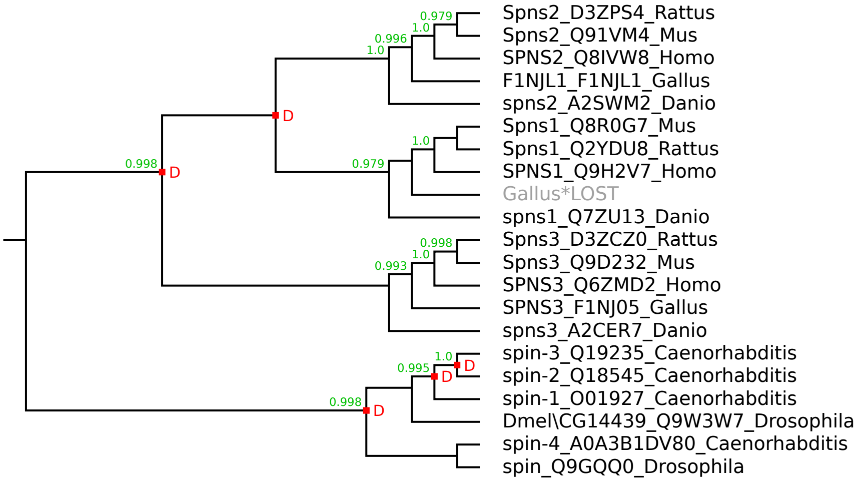

SLC64 family

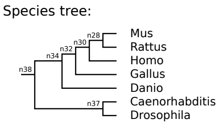

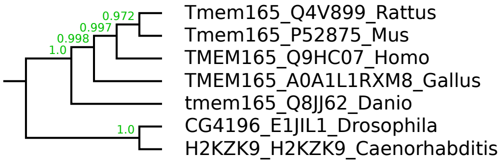

Species tree:

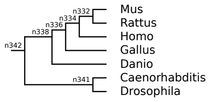

SLC65 family

Warning: this is a large figure that had to be reduced to fit on the page.
Please use the zoom function of your PDF viewer to see the details.

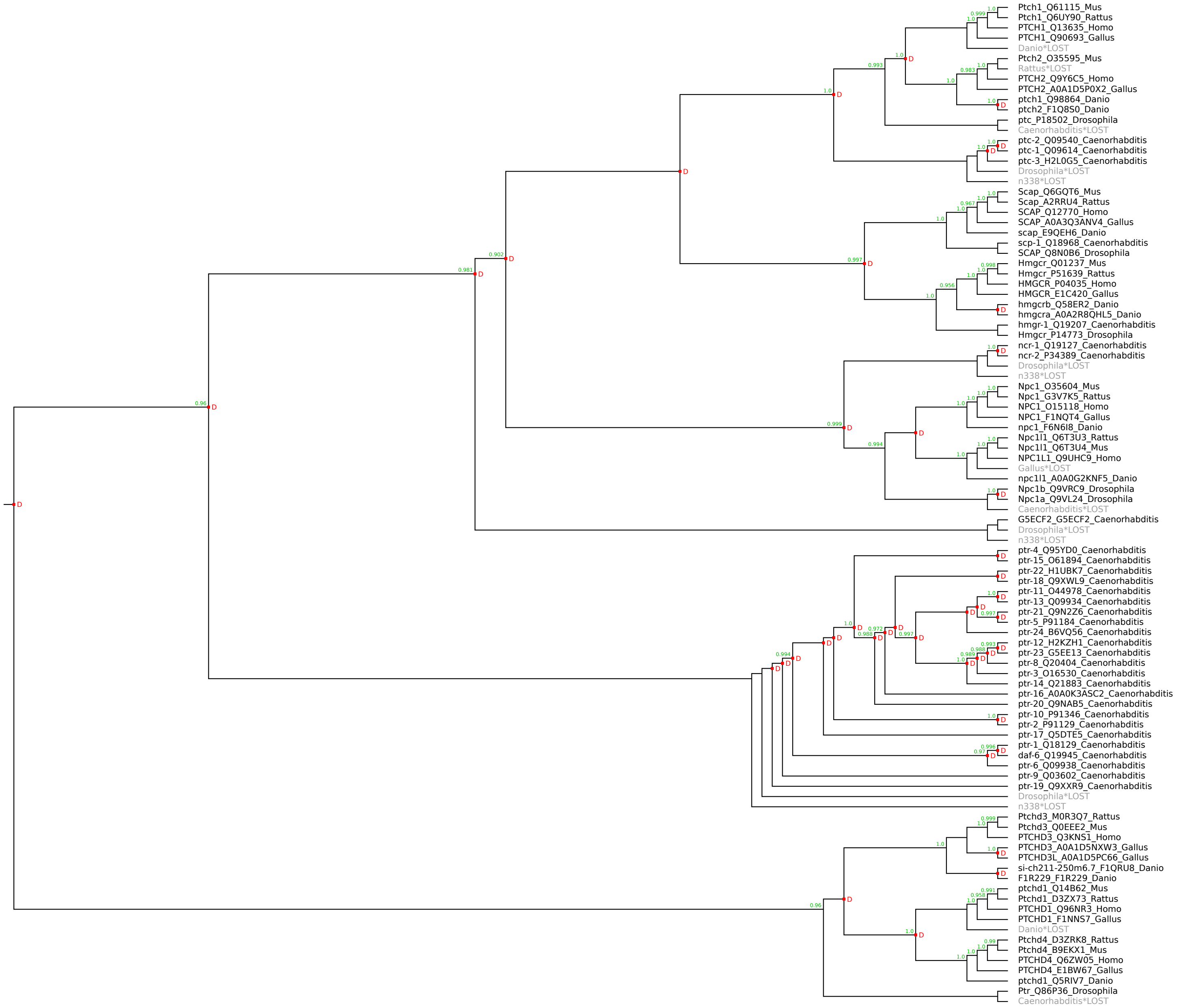

SLC66 family

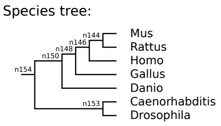

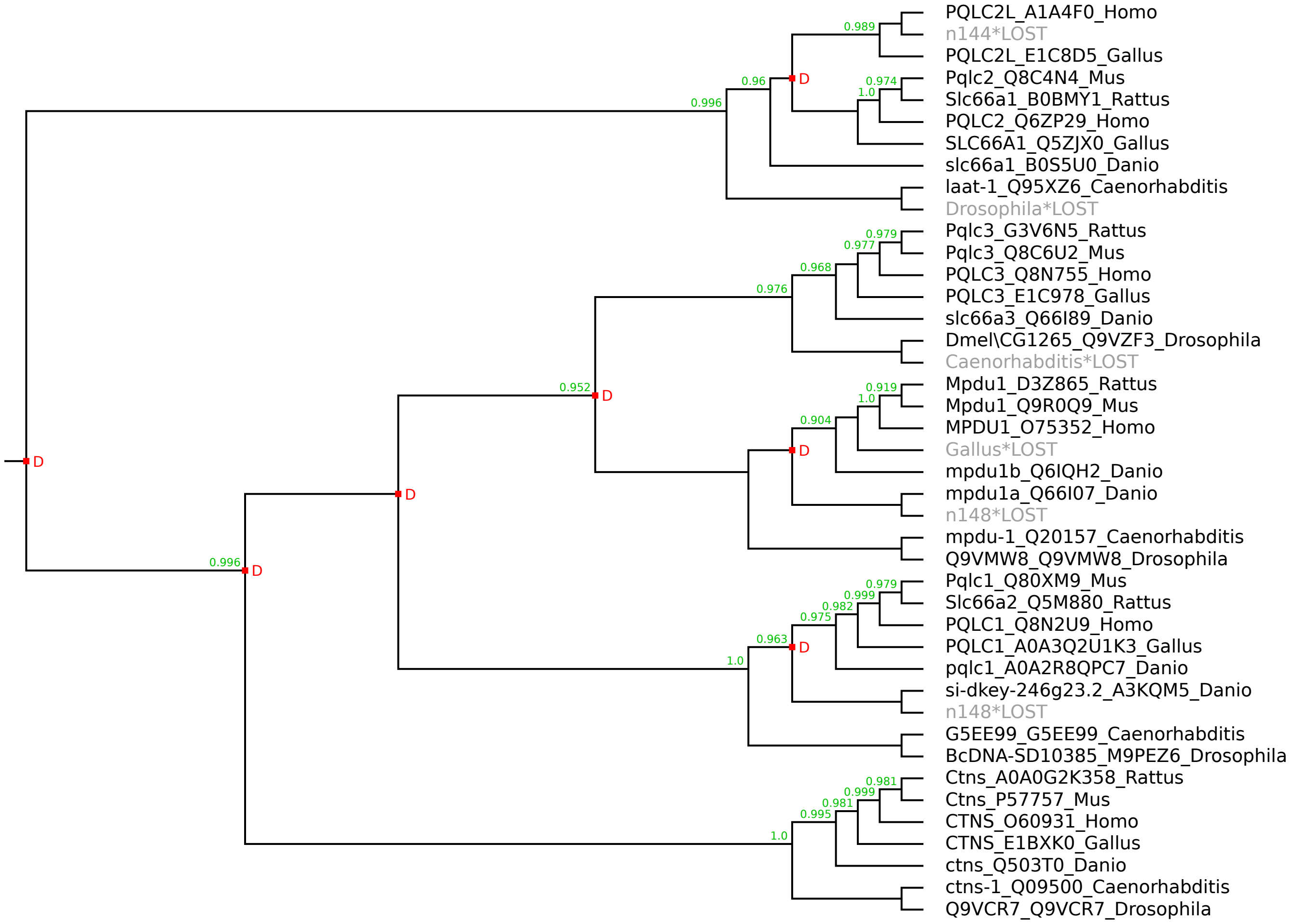

pSLC.5269 family

Species tree:

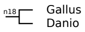

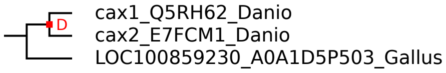

pSLC.7224 family

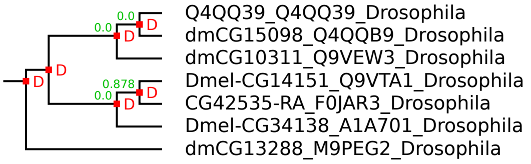

pSLC.ARV1 family

Species tree:

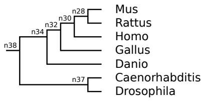

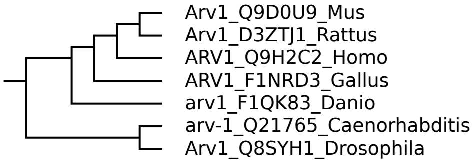

pSLC.Battenin family

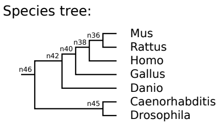

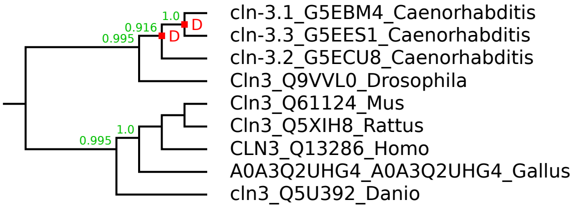

pSLC.CLCN family

Species tree:

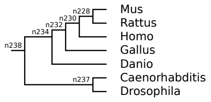

Warning: this is a large figure that had to be reduced to fit on the page.
Please use the zoom function of your PDF viewer to see the details.

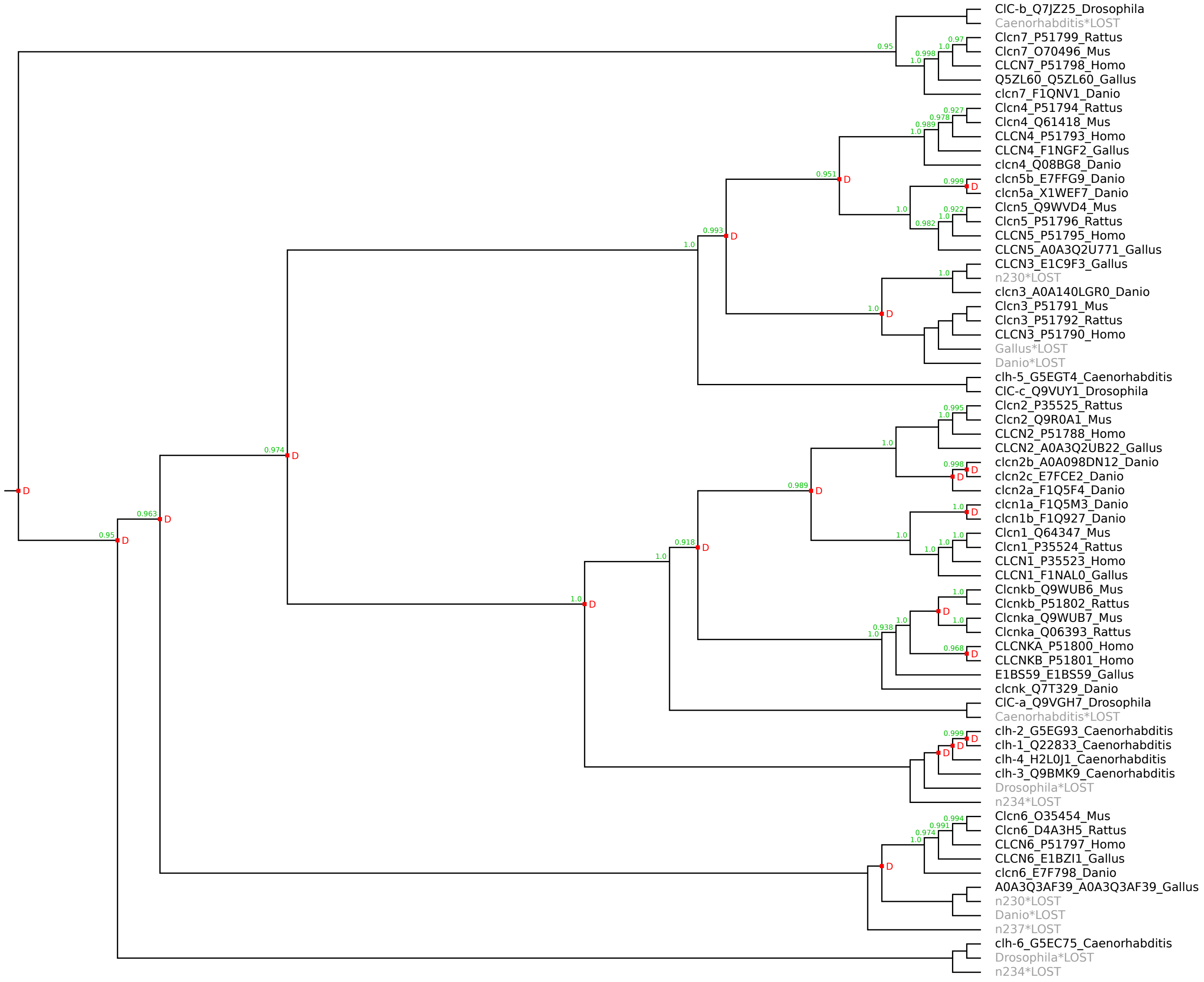

pSLC.CNNM family

Species tree:

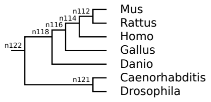

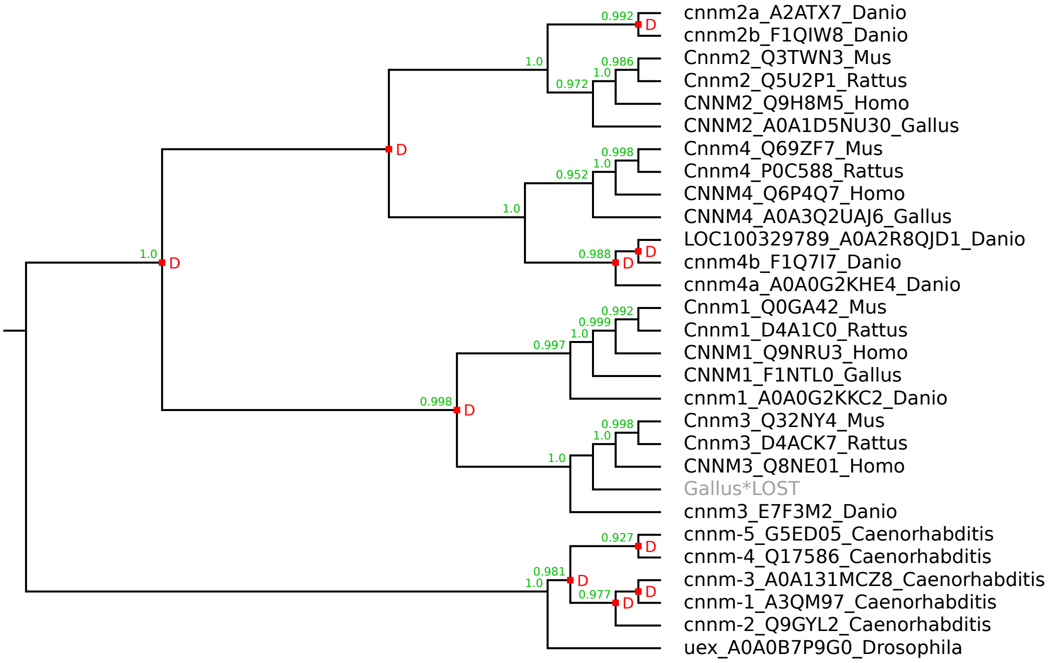

pSLC.CitMHS family

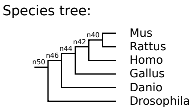

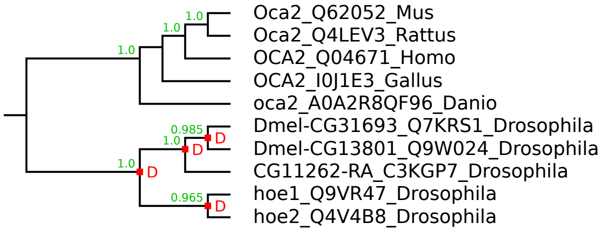

pSLC.Dispatched family

Species tree:

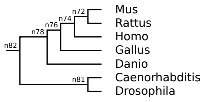

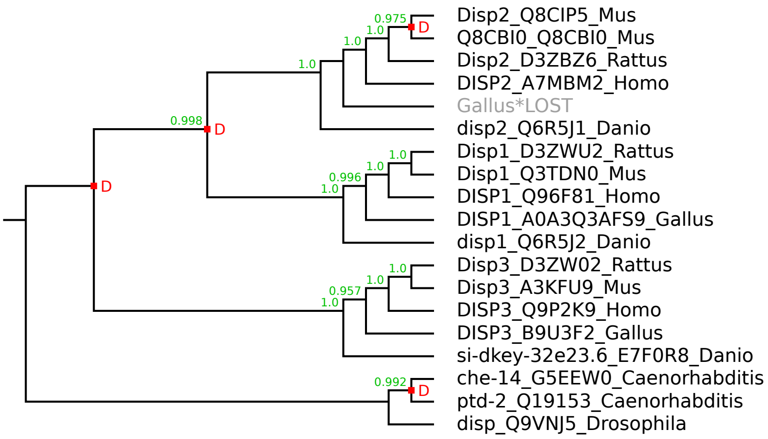

pSLC.GPR155 family

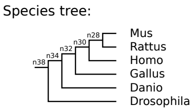

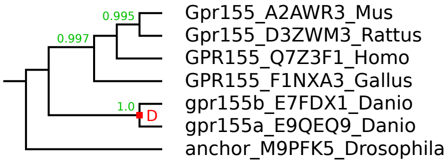

pSLC.LAPTM family

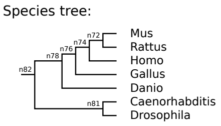

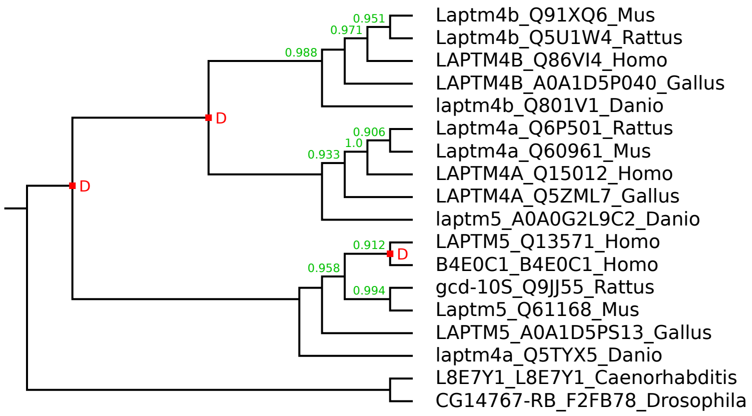

pSLC.LMBR-A family

Species tree:

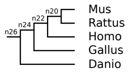

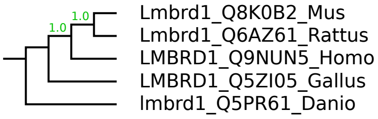

pSLC.LMBR-B family

Species tree:

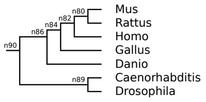

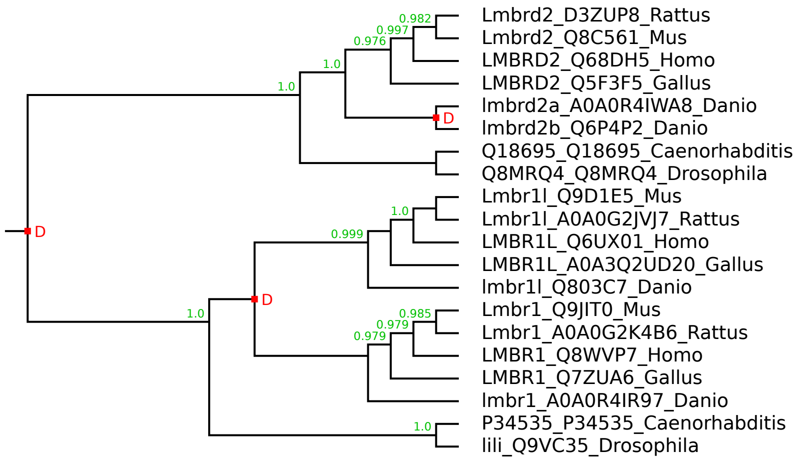

pSLC.MFSD1 family

Species tree:

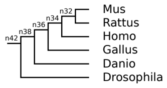

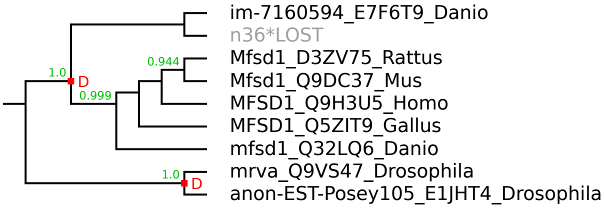

pSLC.MFSD6 family

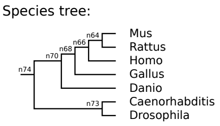

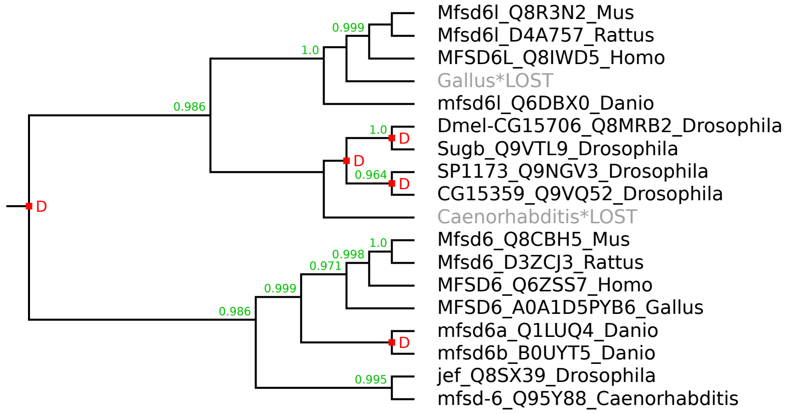

pSLC.RFT1 family

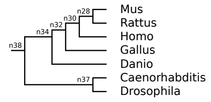

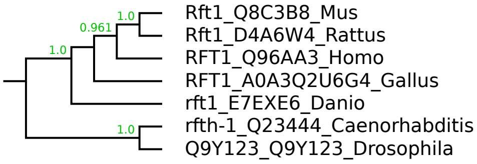

pSLC.SIDT family

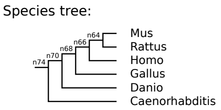

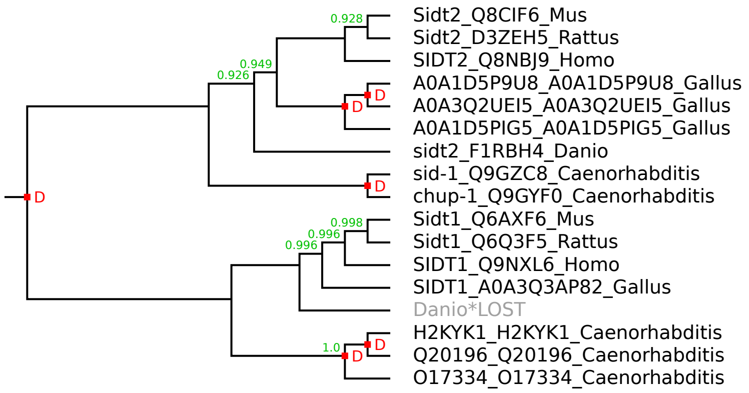

pSLC.STAR family

Species tree:

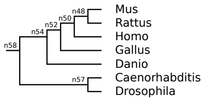

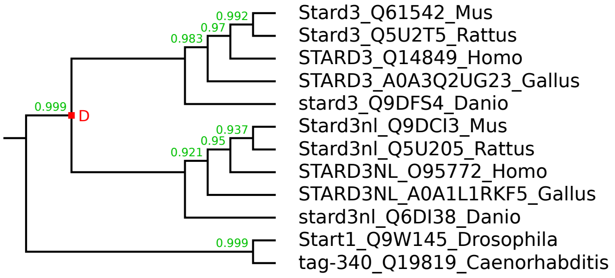

pSLC.STRA family

Species tree:

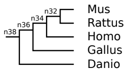

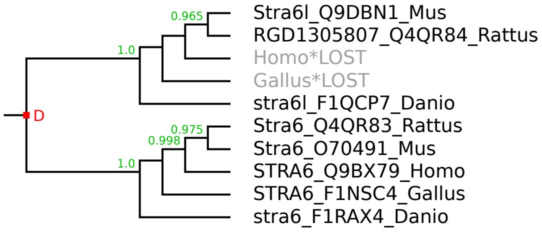

Species tree:

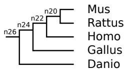

pSLC.TMCO3 family

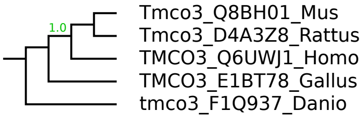

pSLC.TMEM14 family

Species tree:

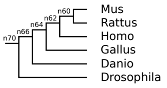

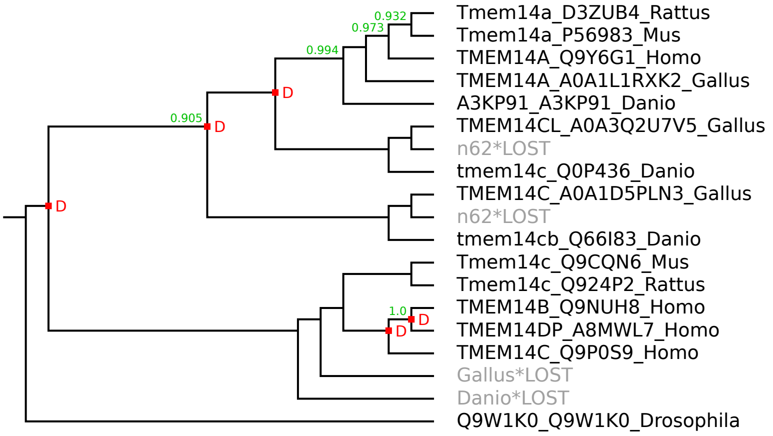

pSLC.TMEM41-64 family

Species tree:

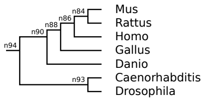

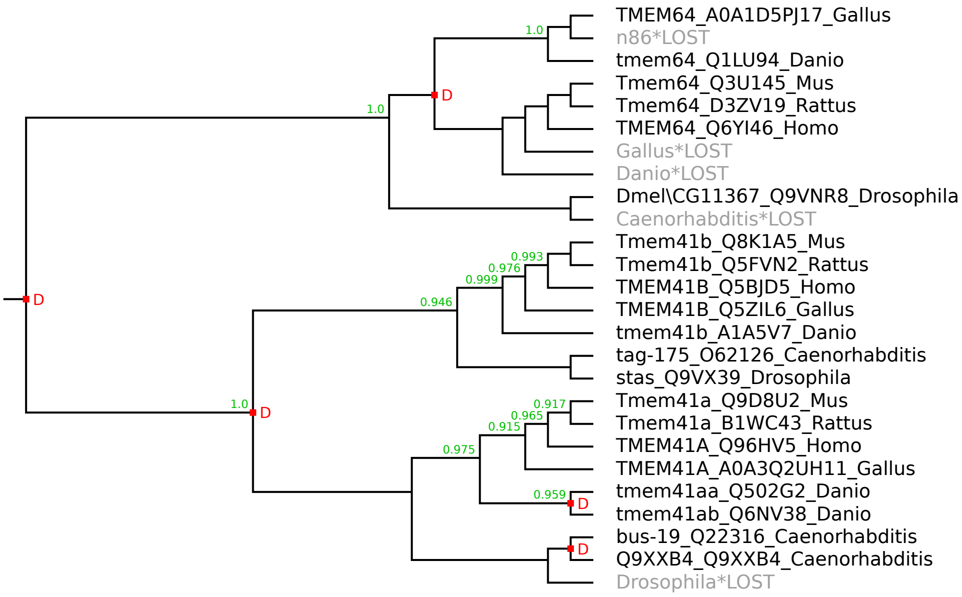

pSLC.TMEM104 family

Species tree:

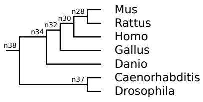

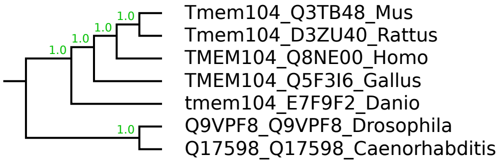

pSLC.TMEM144 family

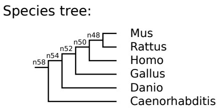

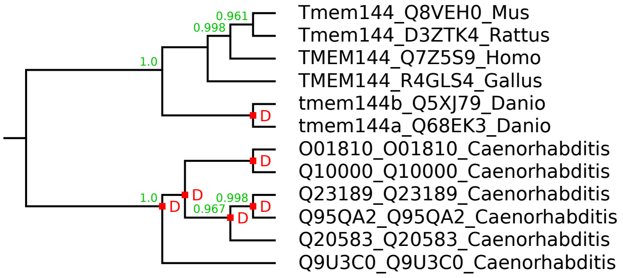

pSLC.TMEM163 family

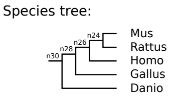

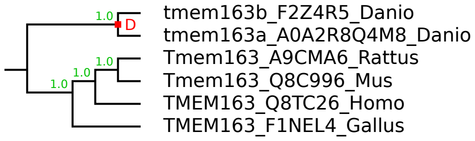

pSLC.TMEM205 family

Species tree:

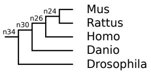

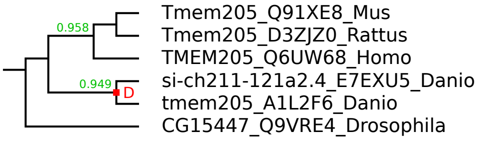

pSLC.TMEM234 family

Species tree:

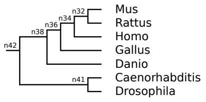

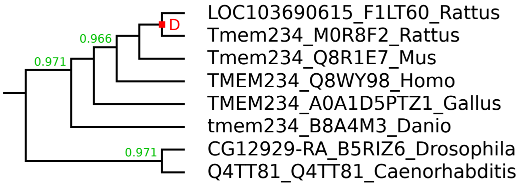

pSLC.TMEM245 family

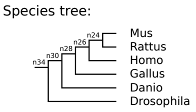

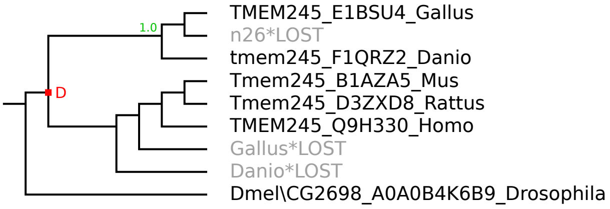

Species tree:

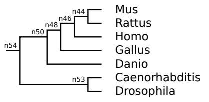

pSLC.TSPO family

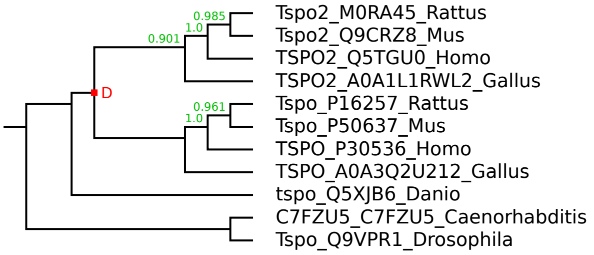

pSLC.UNC93 family

Species tree:

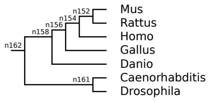

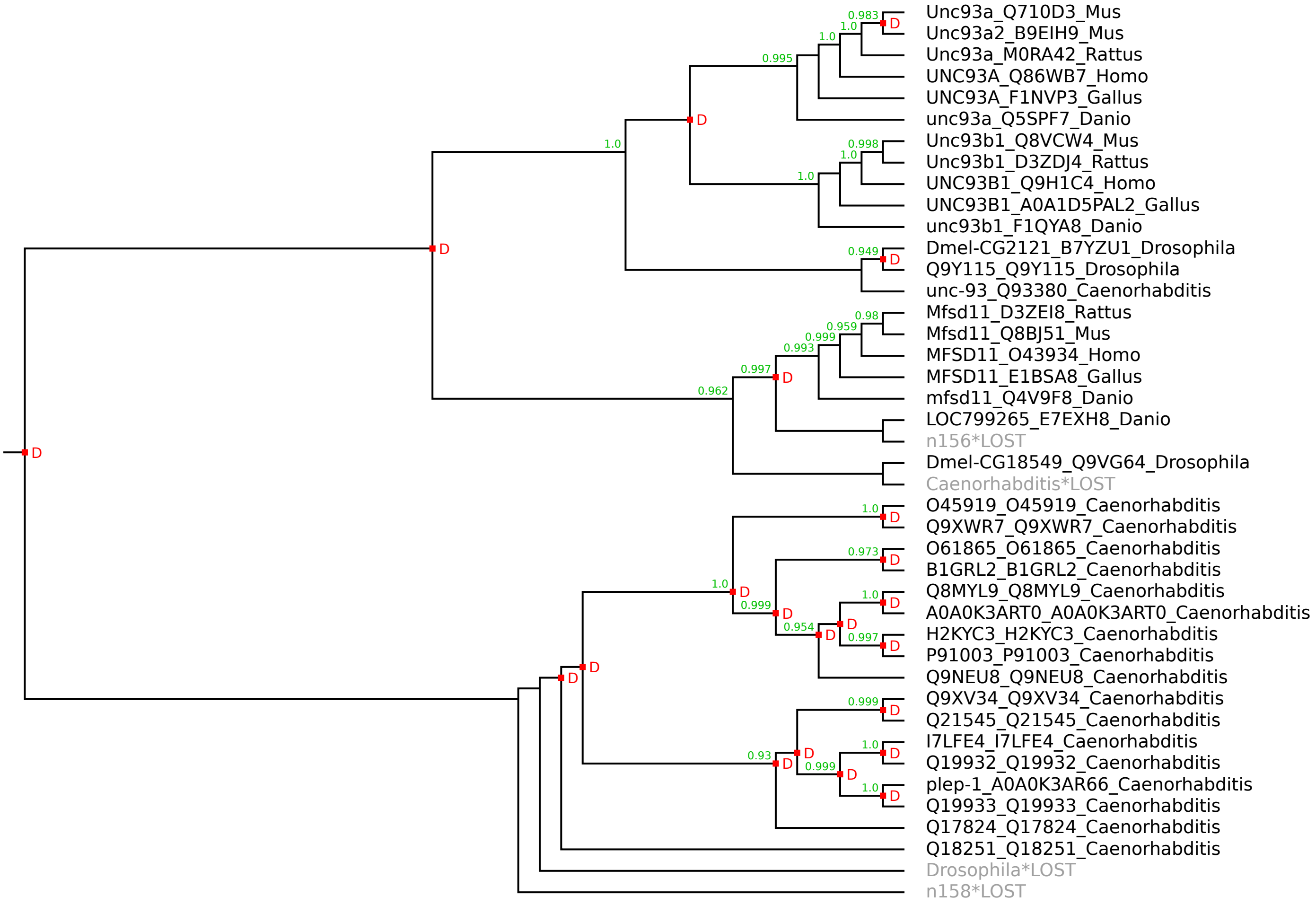

pSLC.XK family

Species tree:

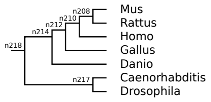

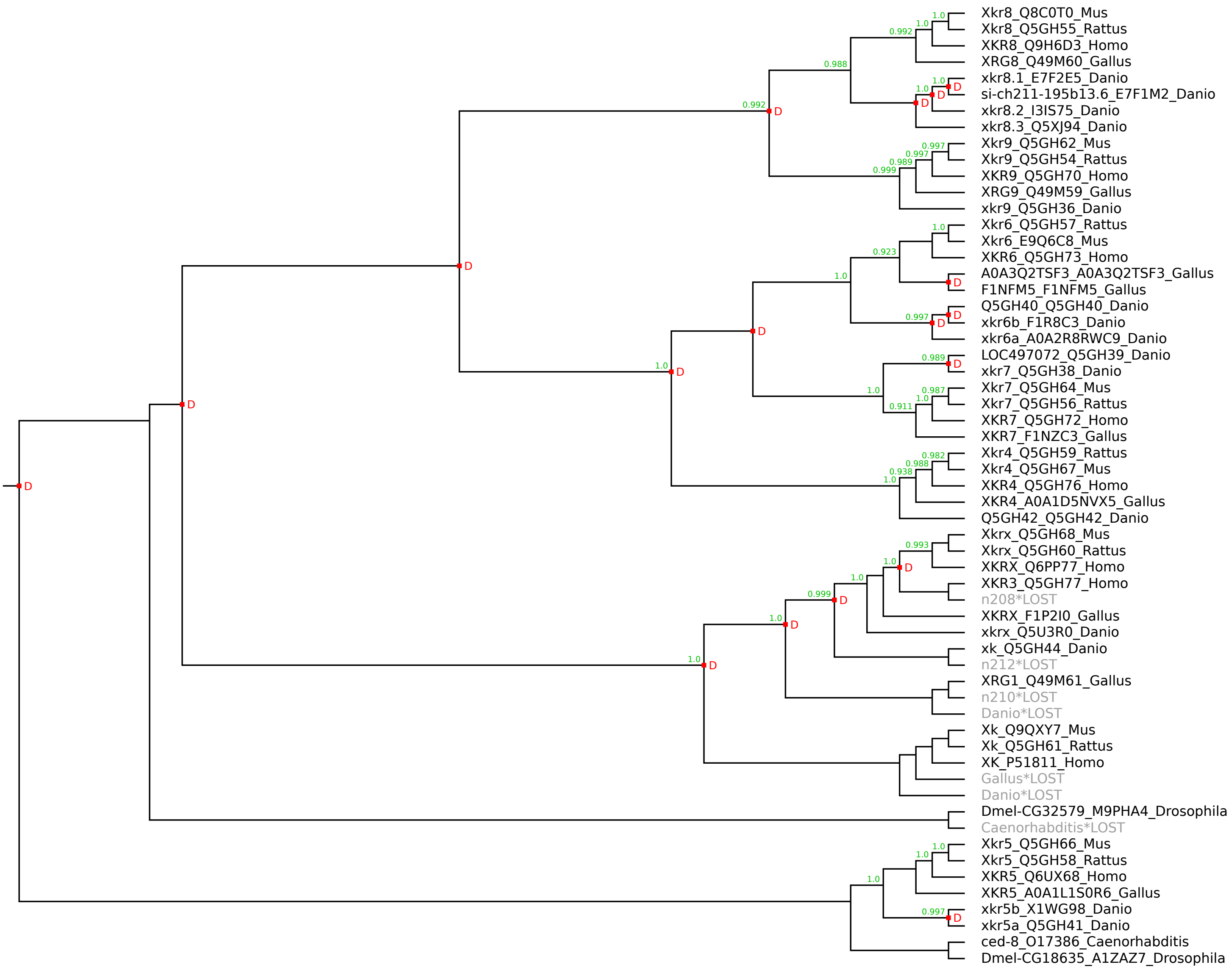

Supplement: S1 File — Trees were generated using multiple alignment by ClustalO, maximum likelihood tree generation by PhyML, followed by tree reconciliation with the species tree using NOTUNG (see Methods). The species tree with internal names of putative ancestor taxa is shown on each page on the upper-right hand corner. The trees are shown as dendrograms and branch lengths are not indicative of evolutionary distance. Each tree leaf corresponds to an SLC-like protein sequence denoting a gene, labels show the gene symbol, UniProt accession and taxon name. Leaves with labels ending with “*LOST” denote putative genes lost in the indicated ancestral species. Red “D” denote gene duplication nodes, normal nodes correspond to speciation nodes. Light green numbers denote branch support values as calculated by NOTUNG. (PDF) [file pone.0271062.s005.pdf]
